# TCP Transcription Factors Involved in Shoot Development of Ma Bamboo (*Dendrocalamus latiflorus* Munro)

**DOI:** 10.3389/fpls.2022.884443

**Published:** 2022-05-10

**Authors:** Kangming Jin, Yujun Wang, Renying Zhuo, Jing Xu, Zhuchou Lu, Huijin Fan, Biyun Huang, Guirong Qiao

**Affiliations:** ^1^State Key Laboratory of Tree Genetics and Breeding, Key Laboratory of Tree Breeding of Zhejiang Province, Research Institute of Subtropical Forestry, Chinese Academy of Forestry, Hangzhou, China; ^2^Forestry Faculty, Nanjing Forestry University, Nanjing, China

**Keywords:** TCP transcription factors, Ma bamboo, expression profiles, bamboo shoot development, transcriptome analysis

## Abstract

Ma bamboo (*Dendrocalamus latiflorus* Munro) is the most widely cultivated clumping bamboo in Southern China and is valuable for both consumption and wood production. The development of bamboo shoots involving the occurrence of lateral buds is unique, and it affects both shoot yield and the resulting timber. Plant-specific TCP transcription factors are involved in plant growth and development, particularly in lateral bud outgrowth and morphogenesis. However, the comprehensive information of the *TCP* genes in Ma bamboo remains poorly understood. In this study, 66 TCP transcription factors were identified in Ma bamboo at the genome-wide level. Members of the same subfamily had conservative gene structures and conserved motifs. The collinear analysis demonstrated that segmental duplication occurred widely in the TCP transcription factors of Ma bamboo, which mainly led to the expansion of a gene family. Cis-acting elements related to growth and development and stress response were found in the promoter regions of *DlTCPs*. Expression patterns revealed that *DlTCPs* have tissue expression specificity, which is usually highly expressed in shoots and leaves. Subcellular localization and transcriptional self-activation experiments demonstrated that the five candidate TCP proteins were typical self-activating nuclear-localized transcription factors. Additionally, the transcriptome analysis of the bamboo shoot buds at different developmental stages helped to clarify the underlying functions of the TCP members during the growth of bamboo shoots. *DlTCP12-C*, significantly downregulated as the bamboo shoots developed, was selected to further verify its molecular function in *Arabidopsis*. The *DlTCP12-C* overexpressing lines exhibited a marked reduction in the number of rosettes and branches compared with the wild type in *Arabidopsis*, suggesting that *DlTCP12-C* conservatively inhibits lateral bud outgrowth and branching in plants. This study provides useful insights into the evolutionary patterns and molecular functions of the TCP transcription factors in Ma bamboo and provides a valuable reference for further research on the regulatory mechanism of bamboo shoot development and lateral bud growth.

## Introduction

Transcription factors play a critical role in plant growth and development and can regulate development by transmitting external environmental factors, mediating hormone signal pathways, and responding to gene regulatory networks in plants (Chen et al., 2021). TCP genes, a kind of plant-specific transcription factors, are typically involved in the plant developmental process, such as seed germination (Zhang et al., 2019a), floral organ development (Wang et al., 2015), leaf morphogenesis (Sarvepalli and Nath, 2011), axillary meristem development (Aguilar-Martínez et al., 2007; Nicolas et al., 2015; Min et al., 2021), and hormone signal transduction (Kosugi and Ohashi, 2002; González-Grandío et al., 2017). The abbreviation “TCP” comes from its earliest discovered members: *Teosinte Branched1* (*TB1*) from maize (*Zea mays*), *CYCLOIDEA* (*CYC*) from snapdragon (*Antirrhinum majus*), and the *PROLIFERATING CELL FACTORS* (*PCF*) from rice (*Oryza sativa*) (Luo et al., 1996; Doebley et al., 1997; Kosugi and Ohashi, 1997). They all have a conserved TCP domain, a basic helix–loop–helix (bHLH) structure, which is primarily related to DNA binding, protein interaction, and protein nuclear localization (Cubas et al., 1999). It can also be divided into Class I (also known as PCF) and Class II according to the characteristics of the conserved domain of TCP proteins. Additionally, Class II can be divided into CIN subfamily and CYC/TB1 subfamily (Martín-Trillo and Cubas, 2010). The most obvious difference between Class I and Class II is the absence of four conservative amino acids in the Class I basic TCP domain. The R domain is rich in polar amino acids such as lysine, glutamic acid, and arginine and is only found in Class II members, which is used to mediate protein interaction (Cubas et al., 1999).

So far, the majority of TCP family members have been determined to play a role in plant growth and development. Previous studies have shown that the *TCP* members of Class I primarily promotes leaf cell proliferation, thereby regulating plant growth and development, and plays an important role in the response to stress (Aguilar Martinez and Sinha, 2013). In *Arabidopsis thaliana, TCP14* and *TCP15* are involved in seed germination by acting downstream of the gibberellin and vernalization pathways (Resentini et al., 2015; Xu et al., 2020). Through binding to the homologous GCCCR elements, AtTCP20 regulates the expression of cyclin and ribosomal protein genes. Furthermore, it acts as a flexible regulator to coordinate growth and division pathways in post-embryonic plant development (Li et al., 2005). Additionally, the downregulation of the expression of *OsPCF6* and *OsTCP21* enhances the tolerance of rice under cold stress by changing the scavenging of reactive oxygen species (Wang et al., 2014). *TCP21* is particularly important during plant growth. Its low expression makes rice more susceptible to rice ragged stunt virus (RRSV) (Zhang et al., 2016; Wang et al., 2021). Overexpression lines of *TCP21* exhibited increased tiller bud length, biomass, and tiller number in rice (Wang et al., 2021). *PeTCP10* is induced by drought and ABA treatment and plays a vital role in plant growth and development and response to environmental stress, which can be seen due to its effects in the evident effects on drought tolerance and the lateral root growth of transgenic lines (Liu et al., 2020). However, the *TCP* members of Class II primarily plays an important role in morphological construction and organ development (Manassero et al., 2013; Sarvepalli and Nath, 2018). Overexpression of *AtTCP1*, the homolog of *CYC*, directly stimulates the expression of the target gene *DWF4* to actively regulate brassinosteroid (BR) biosynthesis, which affects leaf development and leaf shape regulation (Guo et al., 2010; An et al., 2011). It was found that *OsTCP17* (*REP1*), a *CYC* homologous gene in rice, regulates the attributes and development of palea and controls the flower symmetry of the inner and outer lemma axis (Yuan et al., 2009). In wild rice, the *OsTCP15* (*TIG1*) gene is specifically highly expressed on the distal side of the tiller base, which promotes cell elongation by activating the expression of downstream genes such as *EXPA3, EXPB5*, and *SAUR39*, which helps to maintain a large tillering angle (Zhang et al., 2019b).

Branching affects plant morphogenesis and growth to a great extent and determines plant architecture, yield, and ecological sustainability (Richards, 2000; Wang and Li, 2008; Wang et al., 2019). *TB1*, an inhibitory regulator of the development of axillary buds, realizes the transformation from lateral branch growth to apical dominance, transforming teosinte with more tiller numbers into commonly cultivated maize (Doebley et al., 1997; Wang et al., 2019). It has been found in many species due to its conservative function of negatively regulating axillary bud growth (Takeda et al., 2003; Kebrom et al., 2006; Dixon et al., 2018; Shen et al., 2019). *TCP18* (*BRANCHED1, BRC1*) and *TCP12* (*BRANCHEND2, BRC2*) in *Arabidopsis* are the two orthologous homologs of *ZmTB1* in maize, which are highly expressed in axillary buds and negatively regulate the growth of axillary buds, whereas SML 6, 7, and 8 promote branching through transcriptional inhibition of *BRC1* and the non-transcriptional regulation of auxin (Aguilar-Martínez et al., 2007; Wang et al., 2020). Strigolactones (SLs) are a new plant hormone that inhibits the germination of lateral branches and suppresses the growth of tiller buds in rice by regulating the transcriptional level of *OsTB1* (Takeda et al., 2003; Nicolas and Cubas, 2016; Wang et al., 2018). Meanwhile, *OsMADS57* and *OsTB1* jointly regulate the transcription of its target genes *OsWRKY94* and *D14*, realizing the transition between organogenesis and cold adaptation defense in rice at different temperatures (Chen et al., 2018). As wild cucumbers were domesticated, it was found that two light response elements inserted in the promoter region promoted the expression of *CsBRC1*, which directly restrained the auxin outflow from the lateral buds mediated by *PIN3* (encoding auxin transporter) and reduced the production of branches due to the accumulation of auxin in axillary buds (Shen et al., 2019). To date, homologous genes of *TB1* were subsequently identified in numerous gramineous plants such as rice (Takeda et al., 2003), wheat (Dixon et al., 2018), and sorghum (Kebrom et al., 2006). They are very conservative in function, which can cause bud dormancy, and effectively regulate the growth of axillary buds in response to hormones and the external environment which controls the tiller number (Wang et al., 2019).

Bamboo is one of the fast-growing non-timber forest resources and has significant economic, cultural, and ecological value (Zhao et al., 2017). Ma bamboo (*Dendrocalamus latiflorus* Munro) is the most widely cultivated clumping bamboo in Southern China. Its bamboo shoots taste good and are high in nutritional value. Mature bamboo can be used as building materials, decorations, and ornamental planting. Therefore, it is a fast-growing and environmentally friendly clump bamboo species that can be cultivated for its shoots and timber. Bamboo shoots have a longer period of emergence because they are often exposed to the soil surface, which is easily frozen in winter. When cultivating bamboo forests, seasonal shooting and the low germination rate of shoot buds directly affect the yield of bamboo shoots and timber. The formation, growth, and development of bamboo shoots involved in the morphogenesis of lateral branches are unique and affect the yield of bamboo shoots and timber. Therefore, it is important to explore the role of genes related to lateral branch formation in the outgrowth of bamboo shoots, which will help to clarify the molecular mechanism of bamboo shoot development.

Whereas, the TCP family has been characterized in many species, such as *Arabidopsis* (Yao et al., 2007), rice (Yao et al., 2007), sorghum (Francis et al., 2016), and Moso bamboo (Liu et al., 2018), less is known about the TCP transcription factors in Ma bamboo. Recently released genomic data about Ma bamboo, including a representative of hexaploid clumping bamboo (AABBCC, 2n = 6x = 72), allowed us to perform a genome-wide analysis of the TCP transcription factors in Ma bamboo (Zheng et al., 2022). In this study, 66 TCP transcription factor members of Ma bamboo were identified, and the phylogenetic relationship, gene structure and motif information, collinearity, tissue differential expression analysis, subcellular localization analysis, and transcriptional self-activation analysis were analyzed. Additionally, we performed transcriptome analysis of bamboo shoot buds at different developmental stages to clarify the function of the TCP family during bamboo shoot outgrowth. Through the phenotypic observation of transgenic *Arabidopsis*, we concluded that *DlTCP12-C* plays a significant role in controlling the number of branches. Our study reveals the basic information and evolutionary relationship of plant-specific TCP transcription factors in Ma bamboo, clarifies the role of candidate genes in bamboo shoot growth and development by transcriptome analysis, and preliminarily outlines on the function of candidate TCPs, all of which provides valuable insights into future investigations.

## Materials and Methods

### Genome-Wide Identification of Putative *DlTCPs*

To identify TCP transcription factors, the detailed information of *D. latiflorus* genome was obtained through the website (http://forestry.fafu.edu.cn/pub/Dla/). On the Pfam website (http://pfam.xfam.org/), the Hidden Markov Model (HMM) of the conserved TCP domain (PF03634) was downloaded. With a threshold: *e*-values <10^−5^, all putative TCP members with conserved TCP domain were obtained in our protein dataset through the HMMsearch module in SPDE software (Xu et al., 2021). Subsequently, the putative TCP genes (TCPs) were further checked the integrity of its domain through the NCBI (https://www.ncbi.nlm.nih.gov/), InterProScan (http://www.ebi.ac.uk/Tools/pfa/iprscan/), and SMART (http://smart.embl-heidelberg.de/) databases. The genes without complete TCP domain will be manually eliminated. The ExPaSy (https://web.expasy.org/compute_pi/) and Plant-mPLoc (http://www.csbio.sjtu.edu.cn/bioinf/plant-multi/#) were used to predict their molecular weight (MW) and isoelectric point (pI) and subcellular localization, respectively.

### Phylogenetic Tree Construction and Sequence Alignment

All protein sequences of *Arabidopsis*, rice, and Moso bamboo were downloaded from TAIR (https://www.arabidopsis.org/), China Rice Data Center (https://www.ricedata.cn/gene/), and Bamboo databases (http://forestry.fafu.edu.cn/db/PhePacBio/phe/Jbnest.php), respectively. Then, we obtained the sequences of TCP proteins in *Arabidopsis*, rice, and Moso bamboo from previous studies (Supplementary Table S1). To explore the evolutionary relationships, ClustalW was used to perform the multiple sequence alignments between 24 *Arabidopsis*, 22 rice, 16 Moso bamboo, and 66 Ma bamboo TCP proteins with default parameters (Thompson et al., 2003), and MEGA 7.0 was subsequently used to construct a neighbor-joining (NJ) phylogenetic tree with the following parameters: NJ tree method, complete deletion, and 1,000 bootstrap replicates (Kumar et al., 2016). Additionally, the multiple sequences' alignment of DlTCP proteins was performed using DNAMAN software (version 9.0), and the conserved TCP domain regions with 55–60 amino acids were intercepted to further investigate the conservation and diversity. The online tool RNA22 v2 microRNA target detection (https://cm.jefferson.edu/rna22/Interactive/) was used to predict miRNA target sites (Miranda et al., 2006).

### Gene Structure, Conserved Motifs, Chromosome Distribution, cis-Regulatory Element Analysis, Synteny, and Gene Duplication Analysis

The exon–intron structures of *DlTCPs* were mapped using the TBtools software (Chen et al., 2020) based on the obtained coding sequence (CDS) and genomic sequences of Ma bamboo. The online MEME program version 5.4.1 (http://meme-suite.org/tools/meme) was used to identify and analyze the conserved motifs of TCP proteins in Ma bamboo. The 2,000-bp upstream promoter sequences of the *DlTCPs* were submitted to the online program Plant CARE (http://bioinformatics.psb.ugent.be/webtools/plantcare/html/) to search the predicted cis-regulatory elements, and these results were visualized using the online tool Gene Structures Display Server (http://gsds.gao-lab.org/).

MCScanX was used to analyze the duplication events of the *DlTCPs* with the default parameters. The diagram of chromosomal location and synteny relationships was generated by the program Circos (Krzywinski et al., 2009) version 0.69 (http://circos.ca/) based on the information about collinear pairs and genetic location. Meanwhile, collinearity analysis between Ma bamboo and the three other species of *TCPs* was performed using the dual synteny plot module in TBtools. The non-synonymous (Ka) replacement rate and synonymous (Ks) rate were calculated by Ka/Ks calculator to analyze gene duplication events (Wang et al., 2010).

### Plant Materials, Growth Conditions, and qRT-PCR

The seedlings of Ma bamboo were cultured in a culture room at 25°C (16-h light, 8-h dark) with stable humidity. About 6-week-old seedlings in similar growth status were selected to collect the samples of young roots, stems, leaves, and bamboo shoots emerging from the bottom. A total of three biological repetitive samples were collected from each tissue to reduce the experimental error.

Tiangen RNAprep plant kit (Tiangen) were used to isolate total RNA from above-mentioned plant samples. Before dissolving RNA, RNase-free DNaseI (Tiangen, Beijing, China) was used to eliminate any contaminating genomic DNA. Then, 1 μg RNA was reverse-transcribed into first-strand cDNA using Takara PrimeScript First-Strand cDNA Synthesis kit (Takara, Dalian, China). All first-Strand cDNA samples were diluted 5 times and stored at −20°C for real-time quantitative PCR (qRT-PCR) experiments. Gene-specific primers were designed using Primer 5 software and shown in Supplementary Table S2. Glyceraldehyde-3-phosphate dehydrogenase (GAPDH) was used as used internal reference (Liu et al., 2014). qRT-PCR was performed using TB Green™ Premix Ex Taq™ (Tli RNaseH Plus) kit (Takara) with the QuantStudio™ 7 Flex Real-Time PCR instrument (Applied Biosystems). A total of three biological replicates were carried out to eliminate errors. The relative expression level was estimated based on the 2^−Δ*ΔCT*^ method (Livak and Schmittgen, 2001).

### Transcriptome Sequencing and Expression Analysis of *DlTCPs*

The samples used for transcriptome sequencing were collected from Hua'an County, Zhangzhou City, Fujian Province (117°24′-117°35′ *E*, 24°65′-25°02′ *N*) in July 2021. A total of four representative developmental stages of bamboo shoot buds were selected for sampling. In the Stage 1, the largest shoot buds located at the lowest layer of mature bamboo shoots were mainly collected, which were still in the dormant stage. The bamboo shoots were expanding, and the top of the shoots begins to twist and grow upward, which are the main characteristics of bamboo shoots in the Stage 2 (the height is about 8 cm). In the Stage 3, the bamboo shoots grew completely upright, accompanied by the further expansion of the bamboo bodies. The bamboo shoots in the Stage 4 (about 45 cm) have entered the high growth stage, and the height of bamboo shoots is significantly higher than that in the Stage 3 (about 20 cm). After removing the bamboo shoot sheath, the bamboo shoot bud samples around the top 0.4 cm were immediately collected and frozen in liquid nitrogen to prevent RNA degradation. A number of four biological repetitive samples were taken in each stage, and each biological repeat consists of about seven apical buds. The separation of total RNA, quality evaluation, and construction of sequencing library were performed as described in the previous studies (Zou et al., 2021).

Illumina Hiseq 2500 platform (Novogene, Beijing, China) was used for sequencing, and HISAT 2.0.5 software was used to map all clean reads to the reference genome of *D. latiflorus*. The differentially expressed genes (DEGs) were screened by DESeq2 package according to the threshold of fold change ≥1.5 and the adjusted *p*-value < 0.05. Compared with the two groups, the differentially expressed genes (DEGs) were screened by pairwise comparison. The above RNA-seq and bioinformatic analysis were carried out by BioMarker Technologies Illumina, Inc. (Shanghai, China). To further screen the differential genes in the transcriptome, all the differential genes were annotated using Gene Annotation Software for Plants (GFAP) (Xu et al., 2022), which quickly obtained the detailed information of candidate differential expressed genes in the transcriptome.

### Subcellular Localization and Transactivation Activity

To analyze the subcellular localization of candidate genes (*DlTCP5-C, DlTCP7-B, DlTCP9-A, DlTCP12-C*, and *DlTCP23-C*), we designed specific primers to amplify the full-length coding sequence of candidate TCP genes and then inserted them into mGFP fusion expression vector pMDC43 (Supplementary Table S2). Then, the location signal was analyzed in the leaf tissue of *Nicotiana benthamiana* after transforming *Agrobacterium tumefaciens* GV3101 as described in the previous research (Sparkes et al., 2006). The empty vector was used as the control. About 72 h later, the transient expression of GFP fusion protein was observed by LSM900 confocal microscope imaging system (Zeiss, Germany). The nucleus was visualized with mCherry-labeled nuclear markers. Subsequently, the candidate TCP genes were inserted into the pGBKT7 vector to study the transcriptional activity of DlTCP proteins in yeast (Supplementary Table S2). Then, the above recombinant vector, positive control pGBKT7-p53 + pGADT7-T, and negative control pGBKT7 empty plasmid were transformed with lithium acetate method into yeast strain AH109. The transformed strains were cultured on SD medium lacking Trp (SD-Trp) and further selected on defective medium SD-Trp-His-Ade supplemented with X-α-gal for 3–5 days. The transcriptional activation activity was evaluated according to the growth status.

### Cloning of the *DlTCP12-C* Gene and Phenotypic Analysis of Transgenic *Arabidopsis*

The full-length CDS of *DlTCP12-C* was amplified from *D. latiflorus* cDNA and constructed into the binary vector pCAMBIA1300 driven by the CaMV 35S promoter using ClonExpress II One Step Cloning Kit (Vazyme, Nanjing, China) (Supplementary Table S2). Then, *Agrobacterium tumefaciens* (EHA105)-mediated transformation of *Arabidopsis* was used. The transgenic *Arabidopsis* seeds were collected from T1 plants alone, and positive lines were screened on MS medium with 50 mg L^−1^ kanamycin until homozygous transgenic *Arabidopsis* lines of the T3 generation were obtained. The methods of phenotypic observation refer to the previous studies (Li et al., 2021).

## Results

### Identification of *TCP* Genes in Ma Bamboo

A total of 66 TCP members were identified in the *D. latiflorus* genome, among which the number of subgenomes A, B, and C is 22, 20, and 24, respectively. We renamed and classified the DlTCPs according to their subgenomic attribution and chromosomal position. Consistent with *Arabidopsis thaliana*, rice, and Moso bamboo, most DlTCPs belong to the PCF subfamily (35 members), whereas the CIN and CYC/TB1 subfamilies have 21 and 10 members, respectively. The molecular weights (MWs) varied from 13.51 (DlTCP13-C) to 81.80 (DlTCP10-B) kDa, and the lengths varied from 128 (DlTCP13-C) to 768 (DlTCP10-B) amino acid (aa) of DlTCP proteins. The isoelectric point (pI) of the DlTCP proteins varied from 5.16 (DlTCP22-C) to 10.60 (DlTCP9-C). As a typical family of transcription factors, predicted subcellular localization results indicated that the majority of DlTCP proteins were located on the nucleus using the online software Plant-mPLoc. The details of the putative DlTCPs can be found in Supplementary Table S3.

### Phylogenetic Analysis of TCPs in the Four Different Plant Species

**Figure 1:**
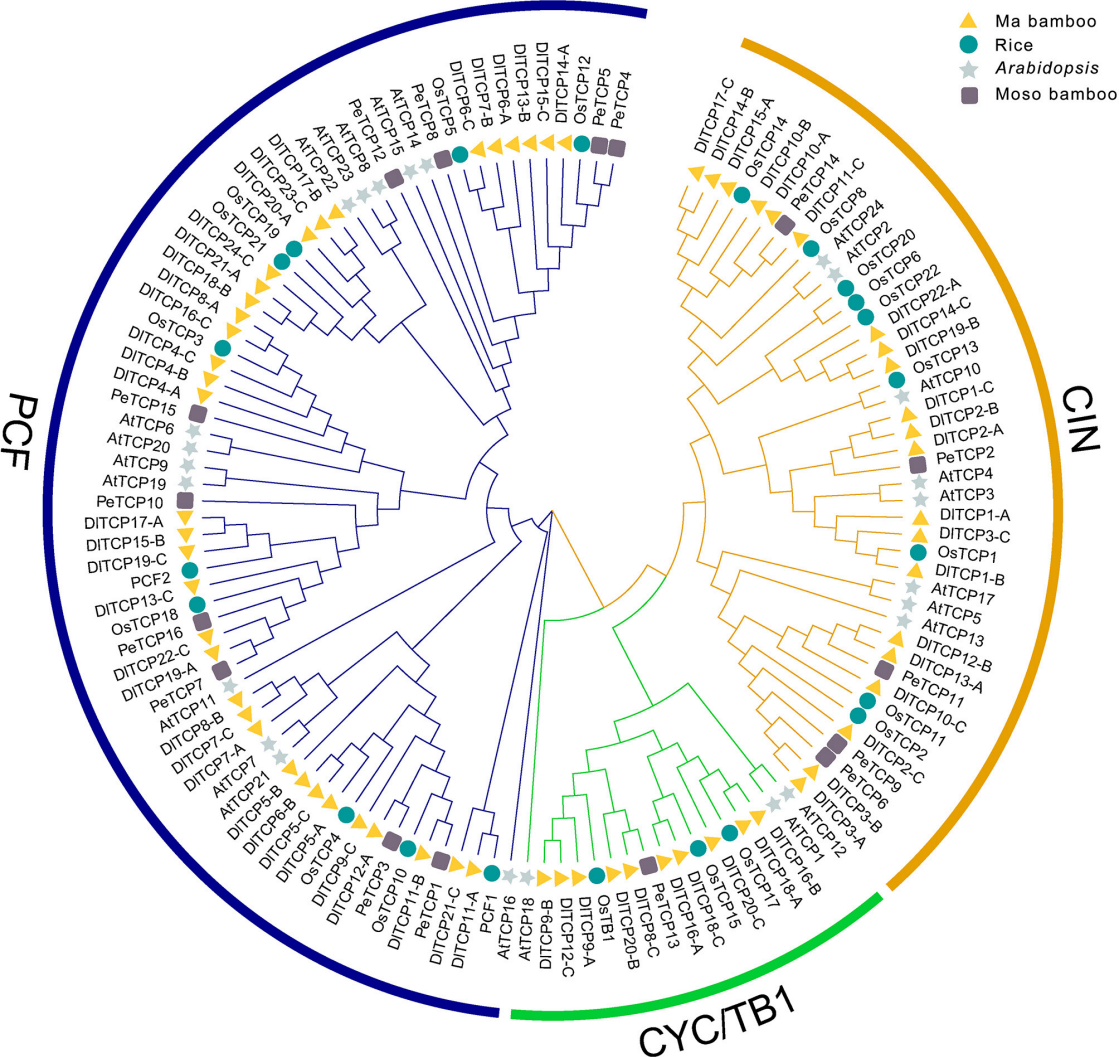
Phylogenetic analysis of TCP members in Ma bamboo, Moso bamboo, rice, and Arabidopsis. MEGA7.0 was used to construct phylogenetic tree by neighbor-joining (NJ) method divided the TCP family into two clades with 1,000 bootstraps replication. Triangle, round, star, and square represent the TCP members of Ma bamboo, rice, *Arabidopsis*, and Moso bamboo, respectively. Different colored geometric shapes were used to mark TCP members of different species.

To further explore the phylogenetic relationship and evolutionary process of the TCP gene family, MEGA7.0 was used to construct phylogenetic trees of the TCP members of Ma bamboo, Moso bamboo, rice, and *Arabidopsis thaliana*. The tree constructed by the neighbor-joining (NJ) method divided the TCP family into two clades with 1,000 bootstraps replication: Class I and Class II. A total of 35 DlTCPs were identified in the Class I (PCF) subfamily, and Class II was further categorized into CIN and CYC/TB1 subfamilies (Figure 1). In addition, the phylogenetic tree showed that the DlTCPs have a close evolutionary relationship between Ma bamboo and Moso bamboo, both of which belong to *Gramineae*. For example, DlTCP11-C and PeTCP14, and DlTCP8-C and PeTCP13 were clustered in the same small subfamily. We also counted the number of TCP family members in different species (Table 1). The results demonstrate that the number of TCP family members in hexaploid Ma bamboo is approximately three times that of diploid plants, which demonstrates the important role of the TCP family during the growth and development of Ma bamboo.

**Table 1:** The number of TCP family members in five plant species.

**Species**	**PCF**	**CIN**	**CYC/TB1**	**Total**	**References**
*Arabidopsis*	13	8	3	24	Yao et al., 2007
Rice	10	9	3	22	Yao et al., 2007
*Sorghum*	9	8	3	20	Francis et al., 2016
Moso bamboo	10	5	1	16	Liu et al., 2018
Ma bamboo	35	21	10	66	

### Gene Structure and Conserved Protein Motifs

**Figure 2:**
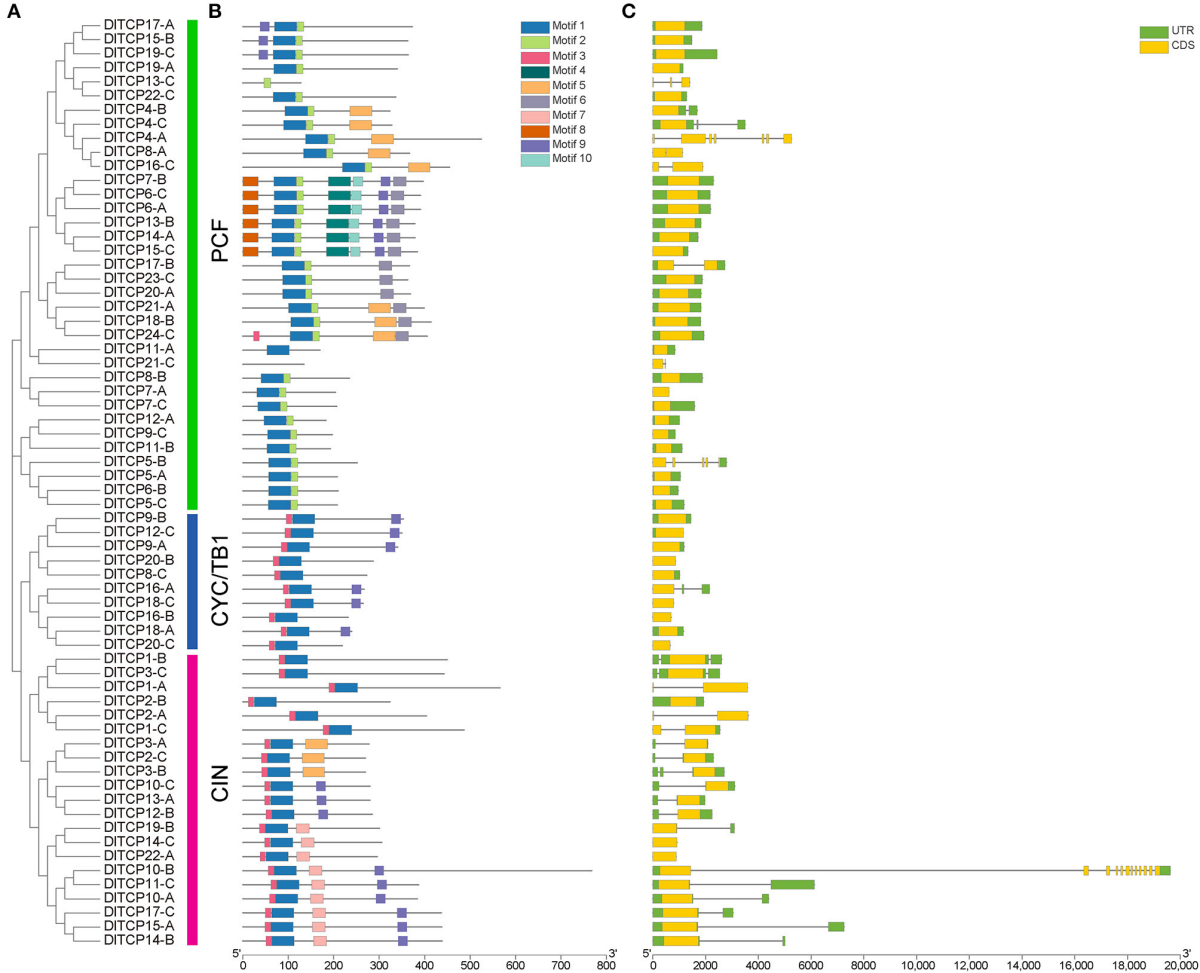
Phylogenetic relationships, motif compositions, and gene structure of TCP genes in Ma bamboo. **(A)** Phylogenetic relationships of 66 TCP members in Ma bamboo. They were further classified into three subfamilies: PCF, CYC/TB1, and CIN. **(B)** Different motif compositions of TCP members in Ma bamboo were detected using MEME. The conserved motifs were represented by boxes with different colors. **(C)** Gene structures of TCPs in Ma bamboo. Green indicates 5′UTR and 3′UTR, yellow indicates exons, and black lines indicate introns.

Gene structure analysis highlighted the differences in conserved domains and motif compositions among subfamilies. However, members of the same subfamily often possess similar structures, for example, motifs 1 and 3 exist in all Class II members (Figure 2). The consistency and difference in this structure can be further confirmed in the results of the multiple sequence alignment of TCP proteins in Ma bamboo. Detailed information of conserved motifs is displayed in Supplementary Table S4. The deletion of four conservative amino acids in Class I members is the most obvious difference compared with Class II (Supplementary Figure S1). Additionally, most TCP members, especially the CYC/TB1 subfamily, showed consistent conservatism with only one exon. In the CIN subfamily, five *DlTCPs* (*DlTCP2-B, DlTCP3-C, DlTCP12-B, DlTCP14-B*, and *DlTCP15-A*) were found to have putative binding sites for *miR319* of Ma bamboo (Supplementary Table S5).

### Chromosome Distribution and Synteny Analysis

**Figure 3:**
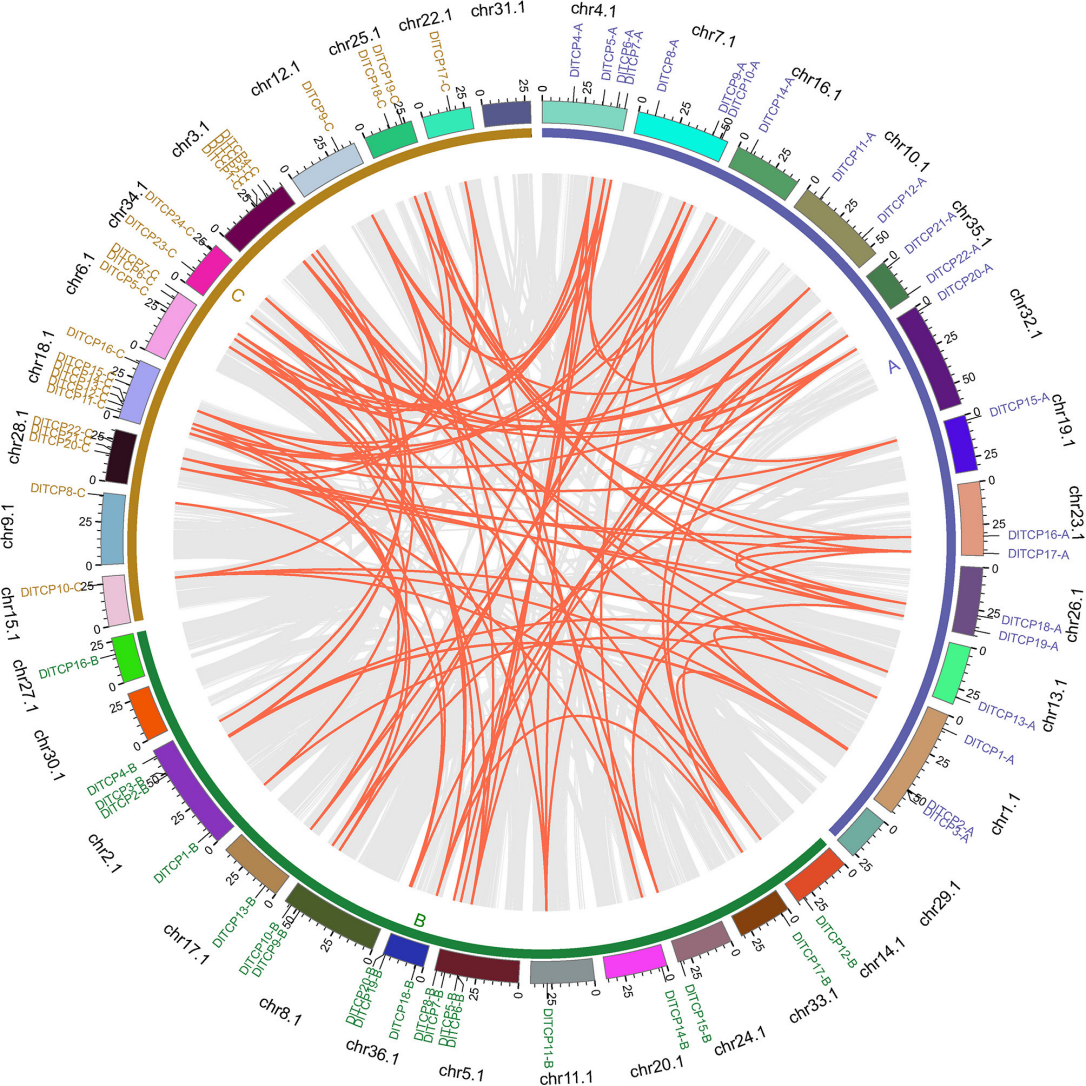
Chromosome distribution and synteny analysis of TCP genes in Ma bamboo. Different chromosomes were showed in different colors. The inner gray lines were used to mark all the collinearity relationships in Ma bamboo and the inner red lines were represented the collinearity relationships of TCP members in Ma bamboo. Gene names of different colors were used to mark the TCP members from different subgenomes.

As expected, synteny analysis demonstrated that the members of the TCP gene family had a very complex colinear relationship in Ma bamboo, suggesting that polyploidization was the main source of the expansion of the TCP gene family (Figure 3). As shown in Figure 3, TCP members have only not been identified on chromosomes 29.1, 30.1, and 31.1, which belongs to the three subgenomes A, B, and C, respectively. The chromosome distribution of TCP members in Ma bamboo was uneven. Chr18.1 contained the largest number of TCP members (6), including 3 PCF members, 1 CYC/TB1 member, and 2 CIN members. Tandem duplication events can drive the renewal of the biological functions of genes. Only one tandem duplication gene pair, *DlTCP5-B* and *DlTCP6-B*, was found in this study.

### Collinearity Analysis

**Figure 4:**
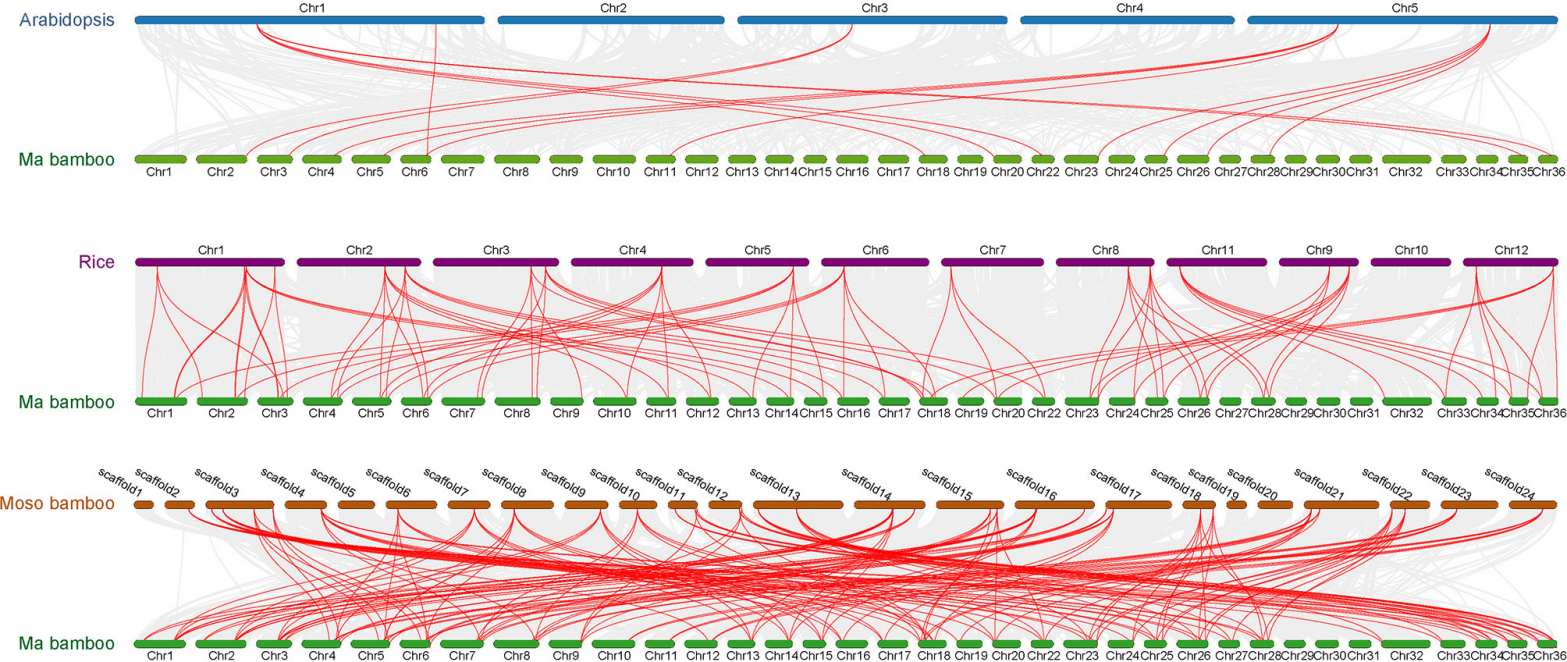
Collinear relationships of genes pairs from Ma bamboo, Moso bamboo, rice, and *Arabidopsis*. Gray lines indicate that all the collinear gene pairs between Ma bamboo and other species, whereas red lines indicate the collinear TCP gene pairs.

The collinearity of TCPs was analyzed in Ma bamboo, *Arabidopsis*, rice, and Moso bamboo by MCscanX. The results demonstrate that Ma bamboo and Moso bamboo, which are both Bambusoideae, had a more conservative evolutionary relationship compared with *Arabidopsis* (Figure 4). However, due to polyploidization, several genes are differentiated during the evolutionary process, producing new gene functions to adapt to the environment. The number of genes with a collinear relationship in Ma bamboo is ~3 times that in rice, indicating that the hexaploid Ma bamboo has a very conservative duplication process during evolution. Homologous gene analysis demonstrated that duplication events occur during genomic evolution. Using phylogenetic tree analysis, 3 putative orthologous gene pairs (*Dl-Os*), 3 putative orthologous gene pairs (*Dl-Pe*), and 14 putative paralogous gene pairs (*Dl-Dl*) were obtained (Supplementary Table S6). The Ks value and Ka/Ks ratio of all putative orthologous and paralogous pairs were calculated to analyze the evolutionary selection and divergence pattern of *TCPs* (Supplementary Table S6). Generally, a Ka/Ks ratio >1, =1, and <1 indicates positive selection, which will be conducive to genetic variation of natural adaptation, neutral selection, and purifying selection to reduce amino acid mutation, respectively. The Ka/Ks ratios of orthologous gene pairs and paralogous gene pairs of TCP members were all <1, indicating that these genes have undergone a strong purifying selection during evolution (Supplementary Figure S2).

### Detection of cis-Regulatory Elements in the Promoter Regions of *TCPs* in Ma Bamboo

**Figure 5:**
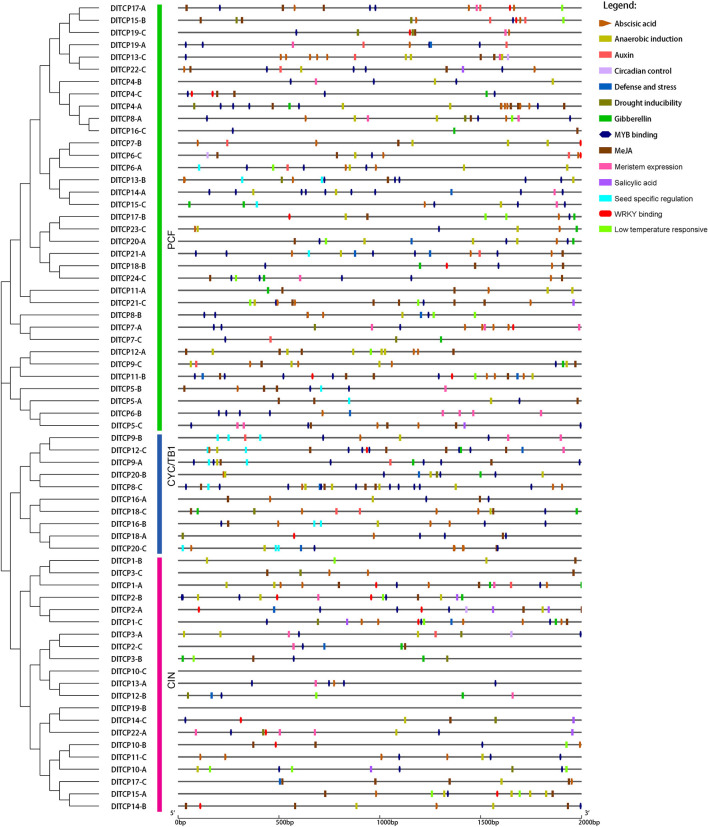
Predicted cis-elements of TCP gene promoters in Ma bamboo. Plant CARE was used to predict and analyze the promoter region of the 2,000-bp upstream of 66 TCP members. Different colored rectangles represented different cis-elements and especial cis-elements were highlighted in different shapes.

To analyze the cis-acting elements of the promoter regions, sequences of the 2,000-bp upstream of 66 TCP members were extracted from the Ma bamboo genome and predicted on the Plant CARE website (Figure 5). A total of 14 cis-acting elements related to hormone response, plant growth and development, and stress have been discovered, including low-temperature-responsive elements, drought-inducible elements, seed-specific regulatory elements, ABA-responsive elements, auxin-responsive elements, and so on. The different cis-elements on promoters may lead to functional differentiation between family members. The number of seed-specific regulatory elements on the promoters of CYC/TB1 subfamily members exceeds that of other members, which suggests that these members have specific functions during seed germination. The results of the phylogenetic tree showed that TCP members that are closely related to each other tend to cluster in a small branch whose promoter region has similar cis-regulatory elements, such as *DlTCP21-A, DlTCP18-B*, and *DlTCP24-C*. This suggests that they have a conserved molecular function. Additionally, the promoter regions of most TCP members have an MYB transcription factor-binding site and WRKY-binding site, especially *DlTCP12-C*, which contains 5 MYB-binding sites and 1WRKY-binding sites.

### Tissue-Specific Expression Patterns

**Figure 6:**
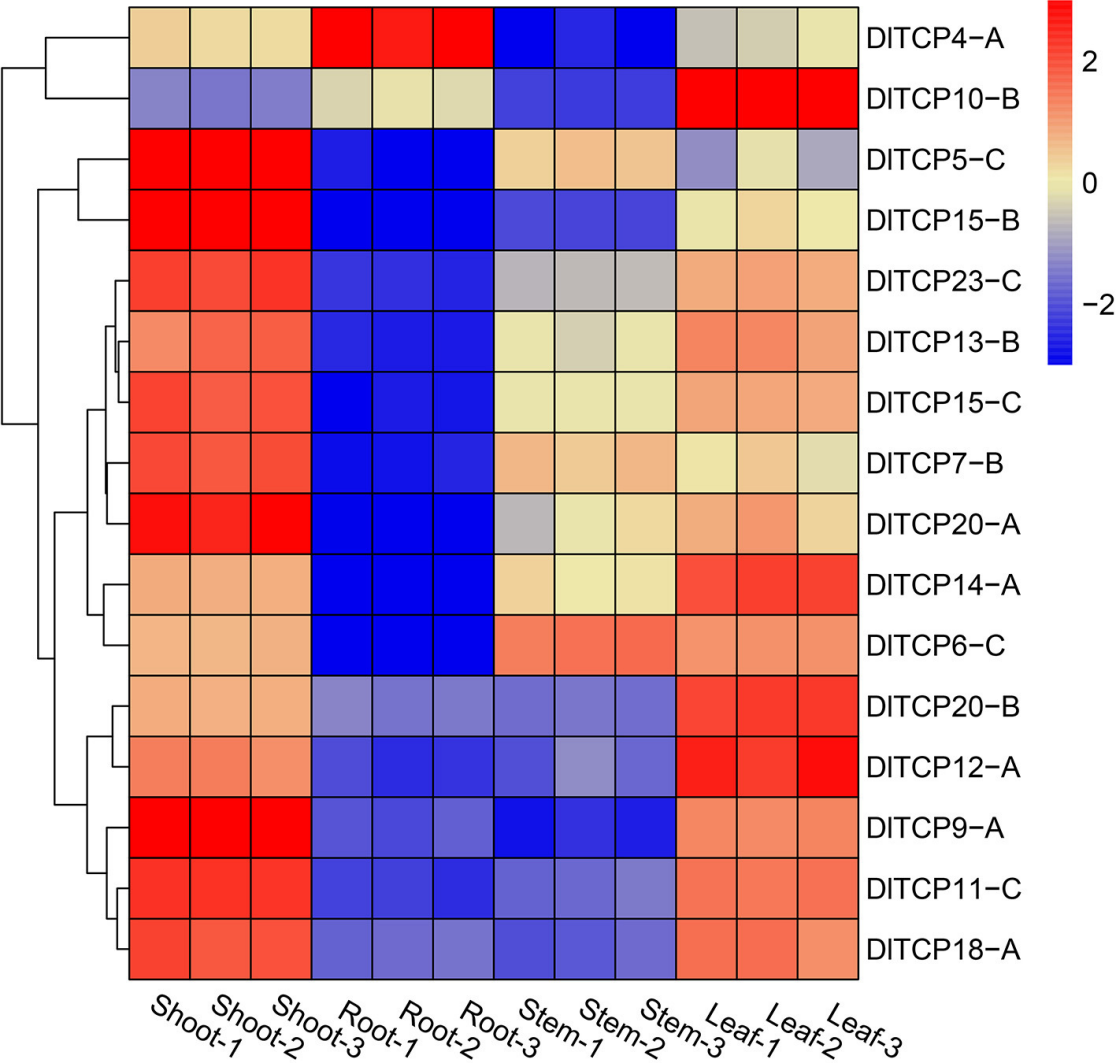
Tissue expression pattern of TCP genes in Ma bamboo. The expression levels of putative TCP members in bamboo shoots, roots, stems, and leaves were normalized and visualized by R (4.0.2). Red and blue represent high and low expression levels, respectively. 1, 2, and 3 represent three biological replicates, respectively.

The tissue expression pattern can deeply reflect the primary role of TCPs, highlighting the need to further study its specific function. According to the above analysis, 16 putative genes were evenly selected from each subfamily of TCP members for tissue expression analysis. The expression levels of putative TCP members were analyzed in bamboo shoots, roots, stems, and leaves of the 6-week-old seedlings (Figure 6). The results demonstrated that TCP family members had obvious tissue expression specificity and were particularly highly expressed in bamboo shoots and leaves. Numerous *TCPs* were highly expressed in shoots, such as *DlTCP5-C, DlTCP15-B*, and *DlTCP9-A*. *DlTCP10-B* was specifically expressed in leaves, whereas *DlTCP-4A* was a transcription factor specifically expressed in the roots. We then attempted to clarify the potential functions of TCP members in shoots by other means.

### Transcriptome Analysis of Shoot Buds at Different Developmental Stages in Ma Bamboo

**Figure 7:**
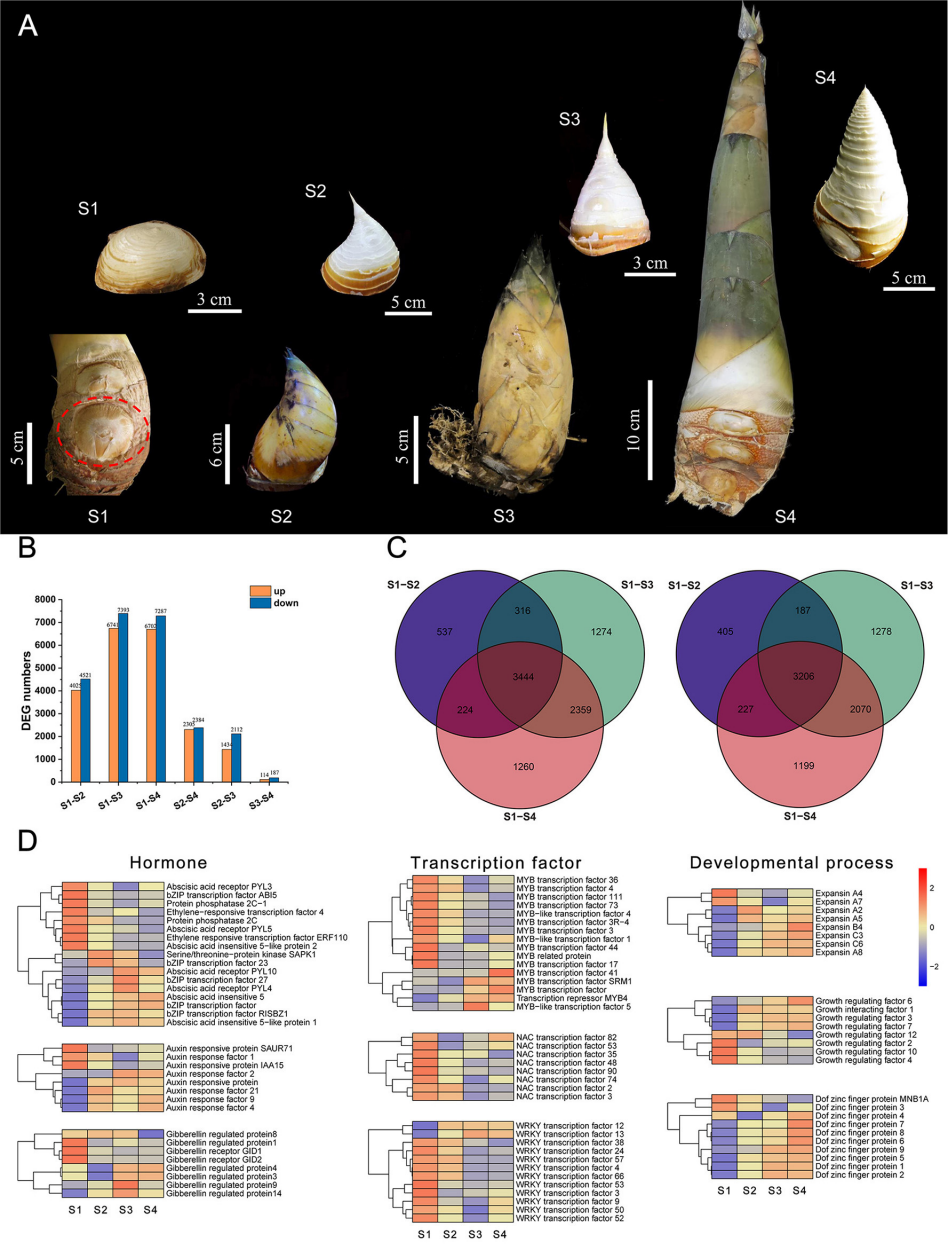
Transcriptome analysis of shoot buds at different developmental stages in Ma bamboo. **(A)** A total of four representative developmental stages of Ma bamboo apical bud were characterized by the length of bamboo shoot. The red dotted line represents the position of shoot buds in S1. A separate scale of each image is shown separately. **(B)** The number of differentially expressed genes between different stages was counted by transcriptome analysis. Yellow and blue represent upregulated and downregulated differentially expressed genes, respectively. **(C)** Venn diagrams of differentially expressed genes in three group. Left and right represent upregulated and downregulated, respectively. **(D)** The expression levels of three kinds of differentially expressed genes in transcriptome data were related to hormones, transcription factors, and developmental process, respectively. Yellow and blue represent upregulated and downregulated differential expression, respectively.

Bamboo shoot buds with a top of ~0.4 cm at four developmental stages were selected for transcriptome sequencing (Figure 7A). We used transcriptome analysis to demonstrate that many growth- and development-related transcription factors were differentially expressed genes, including MYB, NAC, WRKY, and TCP. RNA-seq results indicated that the number of differentially expressed genes (DEGs) in the S1–S2, S1–S3, and S1–S4 groups were higher than in other groups, whereas the number of DEGs significantly decreased between S2, S3, and S4 (Figure 7B). There were only a few differential genes between S3 and S4, which indicates that the morphological structure of the top has been basically developed in later stages of bamboo shoot bud development, making the gene expression very similar. Therefore, we focused on the DEGs in the S1–S2, S1–S3, and S1–S4 comparative groups and selected the common differential genes as the candidate genes. As shown in Figure 7C, 3, 444 and 3,206 DEGs were consistently upregulated and downregulated in the S1–S2, S1–S3, and S1–S4 groups, respectively. The GO enrichment revealed that the DEGs were involved in biological processes, cellular components, and molecular functions (Supplementary Figure S4). Genes related to hormone signaling pathways, transcription factors (MYB, NAC, and WRKY), and developmental processes (expansin, growth-regulating factor, and Dof zinc finger proteins) showed significant differential expression during four developmental stages in Ma bamboo shoots (Figure 7D; Supplementary Table S7).

### Expression Analysis of TCPs in Shoot Buds at Different Developmental Stages

**Figure 8:**
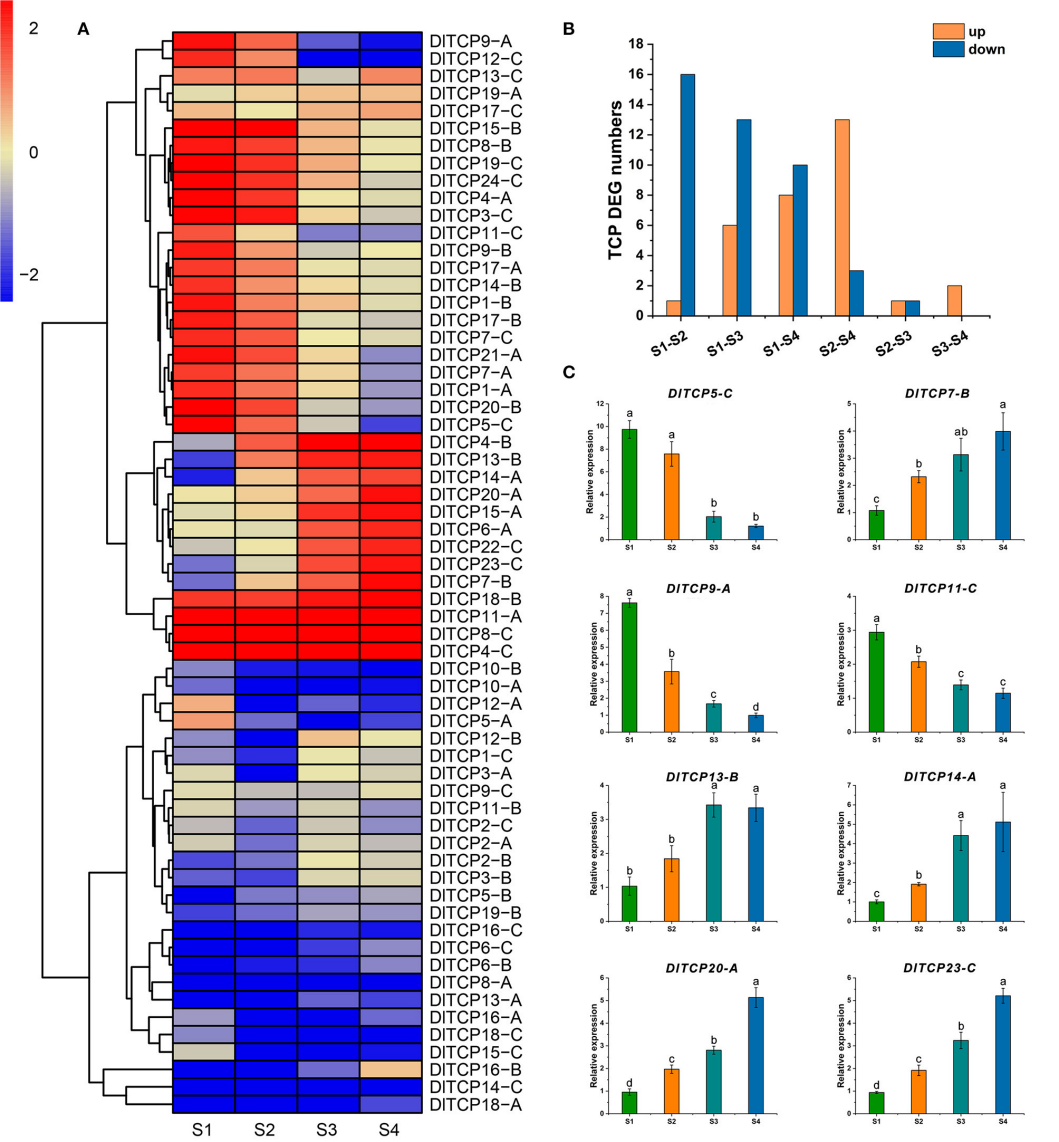
Expression analysis of TCPs in shoot buds at different developmental stages. **(A)** The expression data of TCP family members in different developmental stages of bamboo shoot buds were retrieved from transcriptome analysis. The results were normalized and visualized by R (4.0.2). **(B)** The number of TCP differentially expressed genes between different stages. Yellow and blue represent upregulated and downregulated differential expression, respectively. **(C)** qRT-PCR was used to verify the results of the transcriptome analysis. Error bars were obtained from three replicates. Statistically significant differences between the expression level of different stage were analyzed by Student's *t*-test. Those with different marked letters were represented significantly different: *p* < 0.05.

The expression levels of TCP family members in different developmental stages of bamboo shoot buds were obtained to further explore their roles in shoot bud growth and development using transcriptome data. A total of 29 TCP family members were found as DEGs, and 9 and 20 differentially upregulated and downregulated genes, respectively. The expression levels of *DlTCP9-A* and *DlTCP12-C* belonging to the CYC/TB1 subfamily were significantly decreased during the shoot germination, indicating that they play an important role in the growth and development of bamboo shoots. This is consistent with the molecular function of the *TB1* gene in inhibiting branch growth (Figure 8A). We further counted the differences in DEGs in various stages, and the results indicated that the number of downregulated genes gradually decreased whereas the upregulated genes gradually increased from S1 to S4 in bamboo shoots. This indicates that some members of the TCP family primarily function in the early stage of shoot bud germination and development, while others are highly expressed in the later stages of development (Figure 8B). This emphasized that TCP family members are thoroughly involved in the growth and development of bamboo shoots. Meanwhile, we selected 8 TCP members to verify the reliability of the transcriptome results by qRT-PCR (Figure 8C). GO annotation indicated that TCP transcription factors were involved in growth and development, hormone signal transduction, and stimulus response (Supplementary Figure S3).

### Subcellular Localization and Transactivation Activity

**Figure 9:**
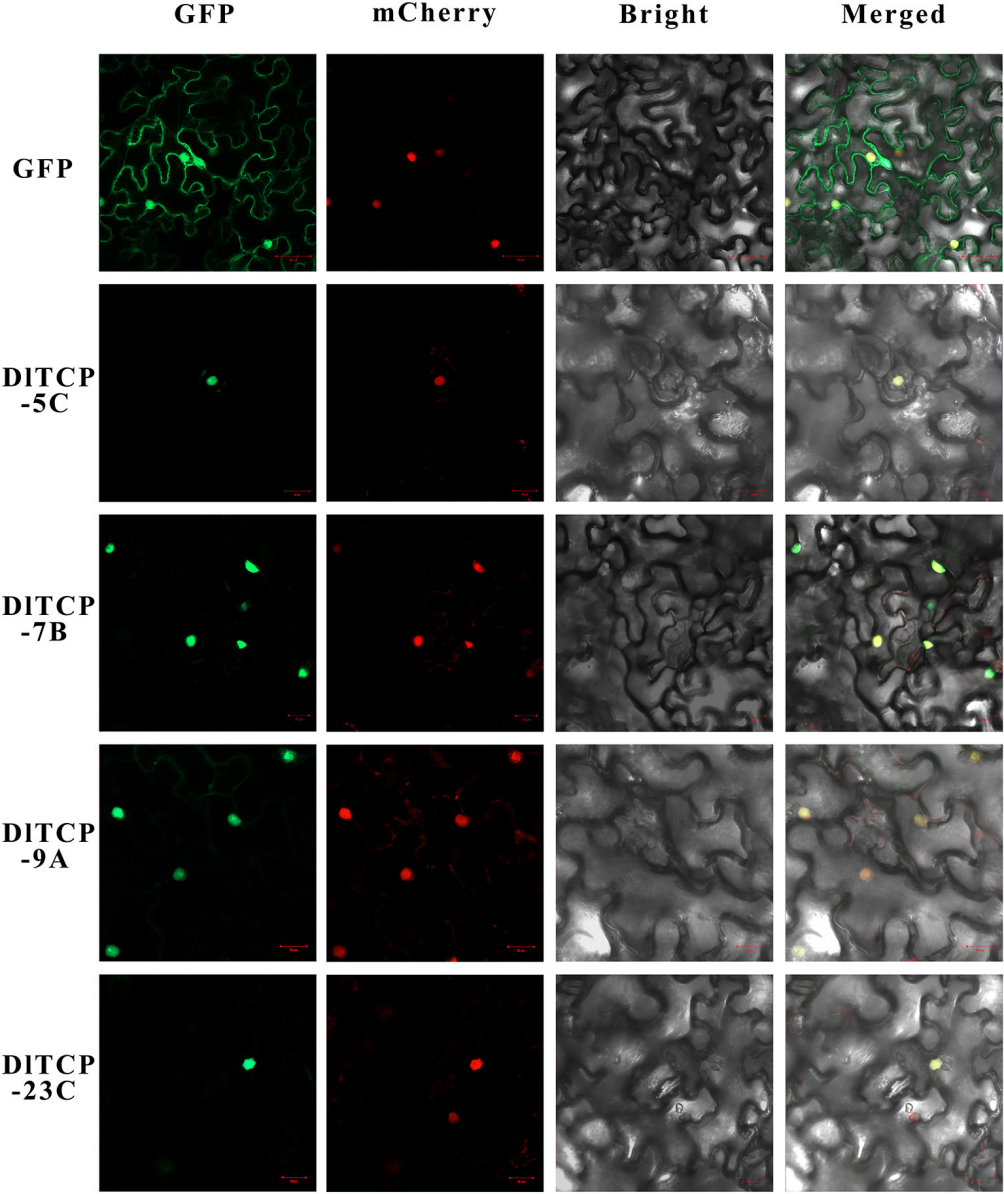
Subcellular localization of four mGFP-fused TCP proteins in Ma bamboo. The four candidate TCP proteins (DlTCP5-C, DlTCP7-B, DlTCP9-A, and DlTCP23-C) and GFP as a control were transiently expressed in *Nicotiana benthamiana* leaves and observed under a fluorescence microscope. The nucleus was visualized with mCherry-labeled nuclear markers.

**Figure 10:**
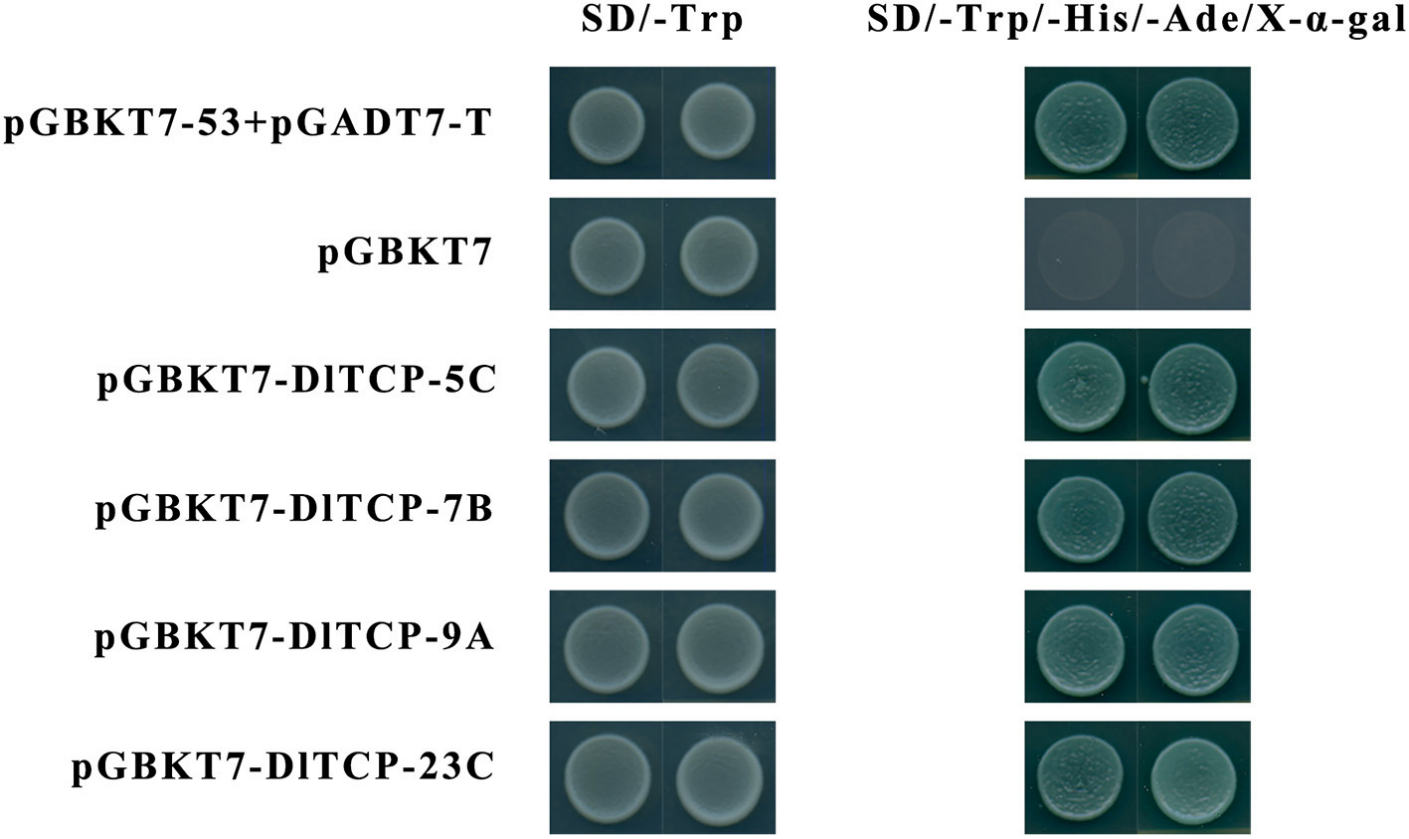
Transactivational analyses of DlTCP proteins in yeast. The positive control pGBKT7-p53 + pGADT7-T, negative control pGBKT7 empty plasmid, and four candidate pGBKT7-DlTCPs plasmids were transformed into yeast AH109, and the strains were further cultured on the yeast medium of SD-Trp and SD-Trp-His-Ade supplemented with X-α-gal to analyze their transactivation activity.

The protein structure of transcription factors is typically composed of four functional domains: DNA-binding domain, transcriptional regulatory domain (including activation domain or inhibitory domain), oligomerization site, and nuclear localization signal. Transcription factors normally function in the nucleus to regulate the expression of their target genes by binding to corresponding binding elements on their promoters. A total of four putative *DlTCPs* highly expressed in bamboo shoots were further selected for subcellular localization and transcriptional self-activation experiment from the 16 genes used for tissue expression analysis. The results showed that *DlTCP5-C, DlTCP7-B, DlTCP9-A*, and *DlTCP23-C* are all nuclear-localized TCP transcription factors (Figure 9). In addition, the yeast transforms with the four candidate genes can grow on the defective medium of SD-Trp-His-Ade supplemented with X-α-gal and turned blue, indicating that *DlTCP5-C, DlTCP7-B, DlTCP9-A*, and *DlTCP23-C* all have transcriptional self-activation activity (Figure 10).

### Cloning and Relevant Analysis of *DlTCP12-C*

**Figure 11:**
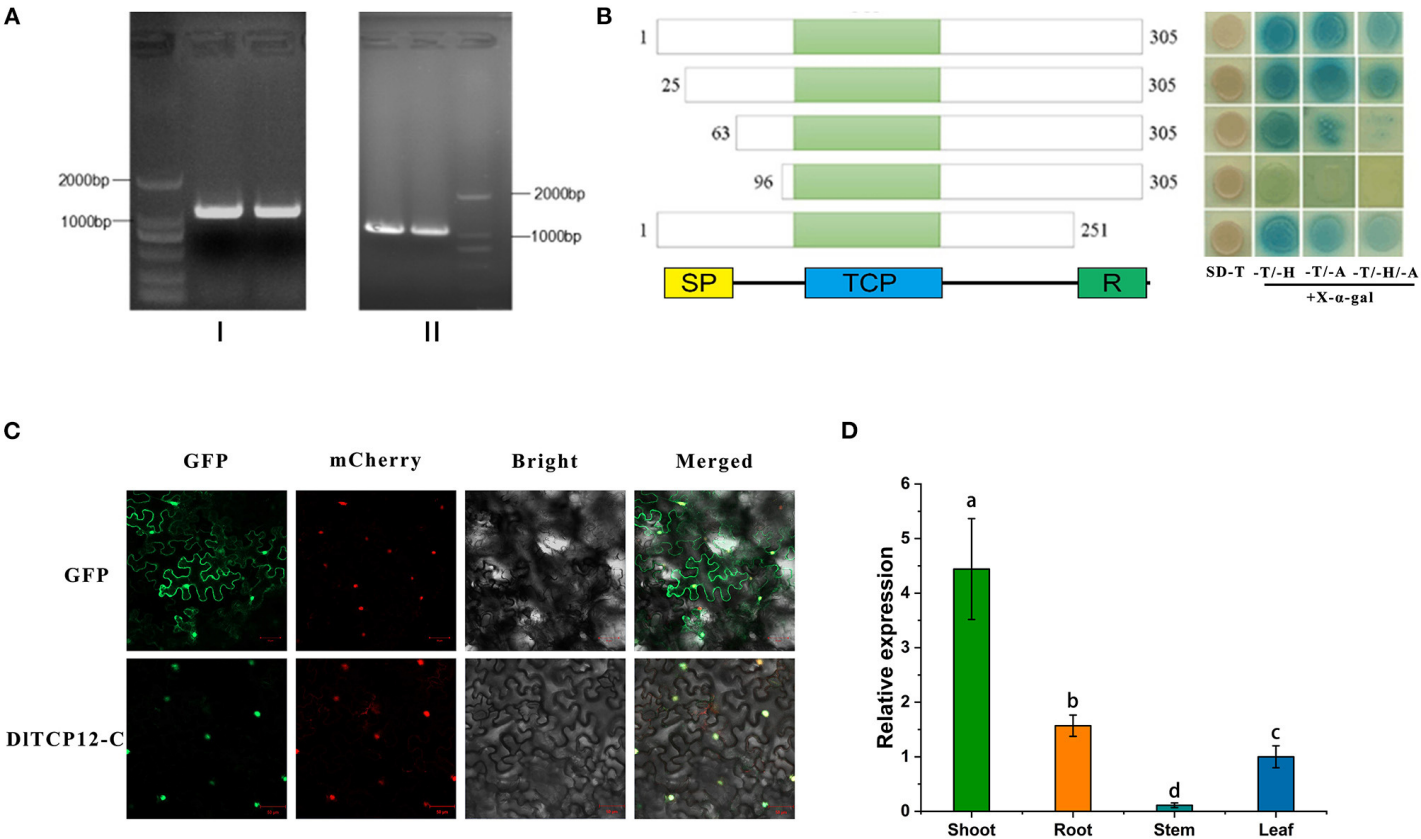
Gene structure, transactivational analyses, subcellular localization, and expression patterns of the *DlTCP12-C*. **(A)** Using genomic DNA (I) and cDNA (II) as template, gel electrophoresis amplification results of *DlTCP12-C*. **(B)** Transcriptional self-activation experiments of five truncated forms of DlTCP12-C, SD-T, -T/-H, -T/-A, and -T/-H/-A represents SD medium lacking Trp, Trp and His, Trp and Ade, Trp, and His and Ade, respectively. **(C)** Subcellular localization of DlTCP12-C protein. **(D)** The expression levels of *DlTCP12-C* in bamboo shoot, root, stem, and leaf; those with different marked letters were represented significantly different: *p* < 0.05.

*DlTCP12-C*, the homologous gene of *OsTB1*, is the key node gene for lateral bud outgrowth, which was significantly differentially expressed according to the transcriptome analysis. This suggests that *DlTCP12-C* could play an important role in the growth of bamboo shoots. Then, the full-length CDS of the *DlTCP12-C* was cloned. The *DlTCP12-C* gene has no intron and contains SP, TCP, and R conserved domains (Figure 11). The original transcriptional self-activating activity was lost after the deletion of 285 bp at the 5' end. The results of the expression pattern and subcellular localization showed that it was a nuclear-localized TCP transcription factor highly expressed in bamboo shoots.

### Phenotypic Assay of the *DlTCP12-C* Overexpression Transgenic *Arabidopsis*

**Figure 12:**
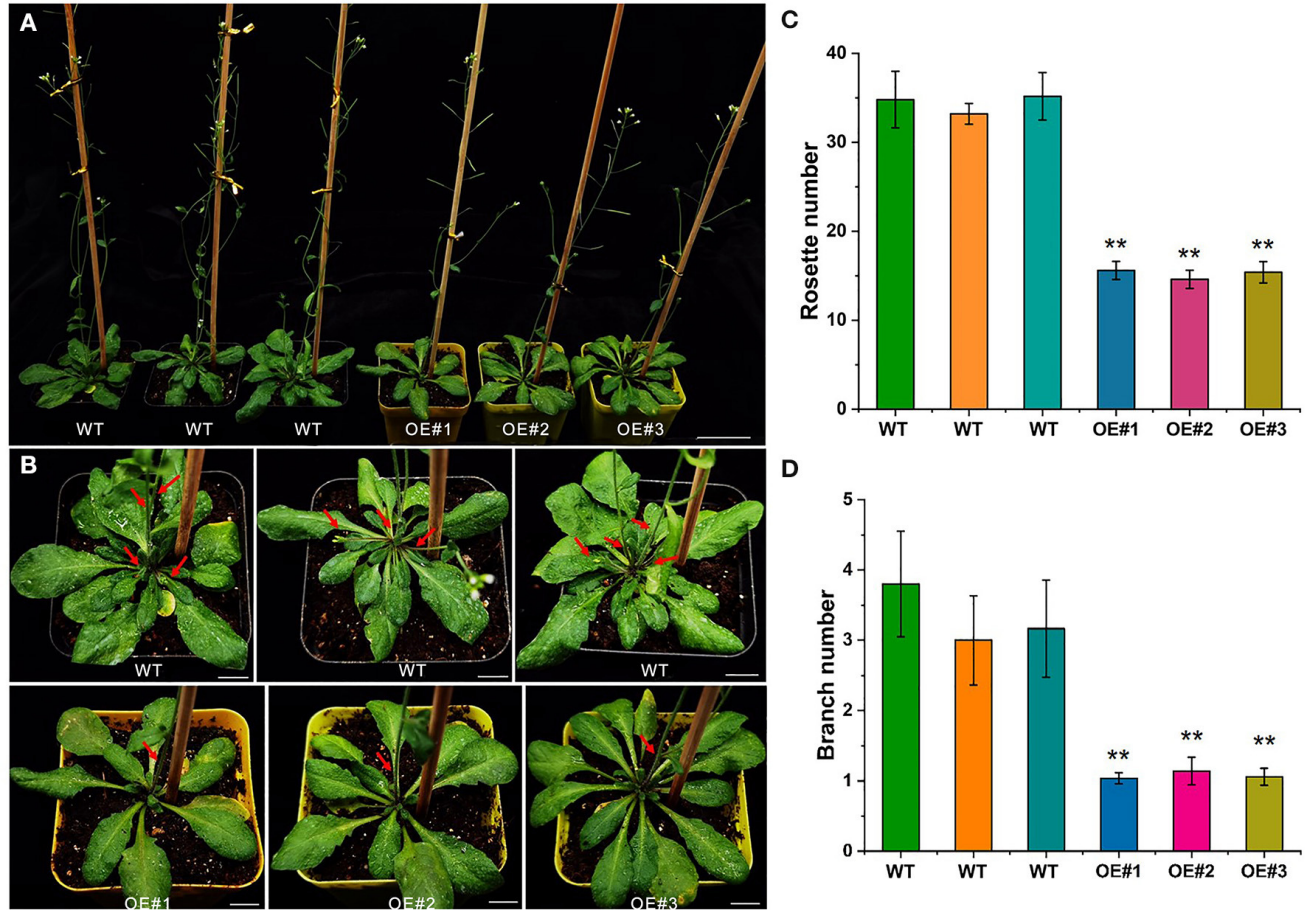
Phenotypic assay of the *DlTCP12-C* overexpression transgenic *Arabidopsis*. **(A)** Branching phenotypes of 35-day-old wild-type and *DlTCP12-C* overexpressing transgenic *Arabidopsis* lines. Scale bar = 4 cm. **(B)** Close-up views of the rosettes of the plants in **(A)**. Red arrow indicates branch. Scale bar = 1 cm. **(C)** Number of rosettes. Error bars were obtained from five replicates. **(D)** Number of branches. Error bars were obtained from five replicates. Significant differences compared with the WT were analyzed by Student's *t*-test: **p < 0.01.

Due to the long period of genetic transformation of Ma bamboo, preliminary functional verification was performed in *Arabidopsis*. Transgenic *Arabidopsis* plants overexpressing *DlTCP12-C* were obtained, and the phenotype of the T3 generation *Arabidopsis* lines was observed after 35 days of culture (Figure 12). Compared with the wild type, the overexpressed lines had significantly fewer rosette leaves and branch numbers. Obviously, no new lateral branches were found in *DlTCP12-C* overexpression transgenic *Arabidopsis* after 35 days of cultivation, whereas there were typically 3–4 branches in WT lines, indicating that *DlTCP12-C* plays an important role in the development of branch outgrowth in *Arabidopsis*. The results of transgene identification and expression detection are shown in Supplementary Figure S4.

## Discussion

As we all know, members of gene families tend to expand through several evolutionary mechanisms, including tandem duplication, large-scale chromosome segmental duplication, and translocation, This leads to the evolution of complex phenotypes (Cannon et al., 2004; McCarthy et al., 2015). In Ma bamboo, numerous genes have undergone triple genome duplication due to whole-genome duplication events (Zheng et al., 2022). Approximately 280 gene families have undergone expansion during their evolution from paleotropical woody bamboos to allohexaploid Ma bamboo, which could be the reason for some of bamboos' unique traits, including their rapid vegetative growth and high biomass (Zheng et al., 2022). In this study, a total of 66 plant-specific TCP transcription factors were identified in Ma bamboo, and the number of genes was about three times that of rice, Arabidopsis, and Moso bamboo (Table 1). In other words, all *DlTCPs* possessed 3–5 homologous genes, indicating that they are preferentially retained during polyploidization. Similarly, there were three closely related homologous genes in the TCP family of hexaploid wheat. However, their cis-acting elements were quite different, which could lead to the sub-functionalization of wheat homologous genes (Zhao et al., 2018). Collinear analysis demonstrated that the main reason for the expansion of the TCP gene family is chromosome polyploidization (whole-genome duplication) and large-scale chromosome segment duplication, which primarily occurs between three subgenomes A, B, and C of *D. latiflorus*, and only a few chromosomes do not possess TCP transcription factors (Figure 3). The TCP family has expanded due to large-scale segment duplication events that also occurred in upland cotton, whereas 74 *GhTCP* genes were identified in the allotetraploid plant upland cotton genome (AADD) (Li et al., 2017). Tandem duplication events, which are important events driving the occurrence of new biological functions, have only been found one time between *DlTCP5-B* and *DlTCP6-B*. This suggests that TCP transcription factors have conservative and irreplaceable functions in Ma bamboo (Shang et al., 2012). The TCP family could have a continually increasing role in the development of the typical hexaploid bamboo species *D. latiflorus*. While they may have functional redundancy, the sub-functionalization of these homologous genes and gene dosage could make Ma bamboo more adaptable during growth and development (He et al., 2022).

Plants have several complex regulatory mechanisms and signal networks, which can quickly perceive the external environment and regulate gene expression to adapt to unpredictable environmental changes and resist several biotic and abiotic stresses in the long-term evolutionary process. Transcription factors can regulate the occurrence of biological processes such as plant morphology, developmental patterns, and stress responses to varying degrees. As the plant-specific transcription factors, the TCP family plays a vital role in plant growth and development. A group of functionally redundant phylogenetic-related class I *TCP* genes (*AtTCP7, AtTCP8, AtTCP22*, and *AtTCP23*) had similar expression patterns in young leaves, regulating leaf development by controlling cell proliferation (Aguilar Martinez and Sinha, 2013). In *Arabidopsis, AtTCP14* directly activates the growth potential of the embryo during seed germination, whose expression level is highest before seed germination (Tatematsu et al., 2008). In addition, the *AtTCP14* mutant was highly sensitive to abscisic acid and gibberellin synthase inhibitors, indicating that *AtTCP14* regulates seed germination by regulating hormone response (Tatematsu et al., 2008; Manassero et al., 2013). The senescence phenotype of *TCP19* and *TCP20* double mutants was significantly enhanced in *Arabidopsis*, and classic genetic and molecular methods have been used to demonstrate that *TCP19* and *TCP20* are involved in controlling leaf senescence in *Arabidopsis*, despite functional redundancy (Danisman et al., 2013). *OsPCF7* is primarily expressed at the tillering stage and plays an important role in the tillering and heading process of rice seedlings. It significantly affects the panicle numbers, the number of filled grains per plant, and the grain yield per plant (Li et al., 2020). *CsTCP3* is induced by gibberellin, photoperiod, and temperature and directly participates in the development of axillary buds by controlling the content of auxin in axillary buds, which affects the number of lateral branches in cucumber (Wen et al., 2020). It can integrate upstream environmental factors and hormone signals and further affect the development of axillary meristem, to adapt the plant architecture to environmental conditions. Biological processes from shoot bud germination to growth is crucial for the growth and development of bamboo (Shou et al., 2020), all of which directly affect the yield of bamboo shoots and timber in Ma bamboo. Therefore, we collected the apical buds of bamboo shoots from four representative developmental stages for transcriptome analysis to find out the relevant gene sets involved in this process (Figure 7A). A total of 29 TCP transcription factors were subsequently found as differentially expressed genes from RNA-seq data. Interestingly, TCP transcription factors exhibited spatiotemporal expression specificity during the development of bamboo shoots (Figure 8A). From the dormancy stage (S1) to the rapid high growth stage (S4), the number of downregulated genes gradually decreased; in contrast, the number of upregulated genes gradually increased (Figure 8B). These results suggested that there is an alternation and switching mechanism in the function of TCP transcription factors to better adapt to the growth and development of bamboo shoots in Ma bamboo. *TB1* is the key node gene for lateral bud outgrowth, which plays a conservative role in many species (Takeda et al., 2003; Dixon et al., 2018; Li et al., 2021). Whereafter, the preliminary functional verification results confirmed the critical role of *DlTCP12-C* in inhibiting axillary bud growth and lateral branch growth in overexpressed transgenic *Arabidopsis* (Figure 12). Future research will verify the biological function and regulation pathway of *DlTCP12-C* in shoot buds development using genetic transformation in Ma bamboo and assess whether there is functional redundancy among other members of the CYC/TB1 subfamily.

Transcription factors have the binding activity of specific DNA sequences or the characteristics of known DNA-binding domains, so they bind to cis-acting elements on the target site to ensure that the target gene is expressed at a specific intensity, in a specific time and place. In our study, a large number of cis-acting regulatory elements related to plant hormone signals, organ development, stress response, *MYB* transcription factors, and *WRKY* transcription factor-binding sites accumulated in the promoter of *DlTCPs*, indicating that TCP transcription factors could act as a central regulatory integrin regulated by environmental factors, hormone signals, and upstream transcription factors to affect plant growth and development. *GhTCP14a/22* is involved in controlling cotton fiber growth through the gibberellin, brassinosteroids, and auxin signal transduction pathways, which play a remarkable role in the development of cotton fiber and are primarily expressed during fiber initiation and elongation (Li et al., 2017). *DWARF27* (*D27*) is a key gene involved in strigolactone synthesis, which can sense strigolactone signaling and activate downstream TB1-like TCP transcription factors by recruiting SCF complexes to stimulate the ubiquitination and degradation of DWARF53 (D53) repressor proteins (Kerr and Beveridge, 2017). Cytokinins and sugars also inhibit the expression of *TB1* (Mason et al., 2014; Patil et al., 2021). Auxin upregulates the expression of MAX3 and MAX4 through the AXR1-AFB-mediated signaling pathway, but downregulates the members of the IPT family, promoting strigolactone biosynthesis, and the inhibition of cytokinin biosynthesis, which further promotes the expression of *TB1* (Nordström et al., 2004; Tanaka et al., 2006). A number of two closely related TCP transcription factors *TCP14* and *TCP15* affect the development of foliage and trichomes, participate in cytokinin-regulated signal pathways, and stimulate the expression of cytokinin-regulated gene RESPONSE REGULATOR 5 through interaction with SPINDLY (SPY) (Steiner et al., 2012). Jasmonic acid is a kind of plant hormone of lipids (oxylipins), which is involved in plant development, abiotic stress response, and the interaction between plants and microorganisms. *AtTCP4* reportedly directly targets *LIPOXYGENASE2* (*LOX2*), encoding a chloroplast enzyme gene involved in α-linolenic acid biosynthesis and jasmonic acid synthesis, and is involved in the regulation of jasmonic acid biosynthesis and leaf development (Vick and Zimmerman, 1983; Danisman et al., 2012). The LsAP2 transcription factor further regulates leaf morphology in lettuce by inhibiting the activity of the CIN-like TCP transcription factor (Luo et al., 2021). The TCP-mediated complicated hormone signal regulatory network further emphasizes the important role of TCP in affecting plant growth patterns and biological processes, and more in-depth research is needed to clarify the pathway of TCP transcription factors. Our transcriptome and promoter element analyses indicate that TCP transcription factors regulate the expression of target genes through transcriptional regulation and hormone signals, which affect related biological processes in plants. Recent studies have made clarified the mechanism of the direct targeting regulation of TCP transcription factors by miRNA. Some TCP members directly targeted by *miR319* are widely involved in plant hormone signal transduction, leaf development, vascular formation, and response to abiotic stress (Fang et al., 2021). Comprehensive analysis of catechin metabolism profiles and *TCP* gene expression profiles in different plant tissues at different developmental stages indicated that the *CsmiR319b*/*CsTCP3-4* module was not only related to shoot tip development, but also played a potential role in catechin biosynthesis in tea plants (Yu et al., 2021). Genetic and molecular analyses indicated that *PtoTCP20*, the direct target gene of *miR319a*, regulated the proliferation of vascular cambium along with *PtoWOX4a* and promoted the differentiation of secondary xylem by activating the transcription of *PtoWND6*, thereby regulating the secondary growth of *Populus tomentosa* stem (Hou et al., 2020). A total of five *TCP* genes were found to contain miR319 directly targeted binding sites in 3′ UTR (Supplementary Table S6). These miR319-targeted *DlTCPs* were the members of the CIN subfamily. The regulation mechanism of DlTCPs related to the bud growth of bamboo shoots requires further study.

In short, this study identified 66 plant-specific TCP transcription factors in the *D. latiflorus* genome using bioinformatics and analyzed their evolutionary relationship, duplication events, promoter cis-elements, tissue expression patterns, subcellular localization, and self-activating transcriptional activity. Transcriptome analysis of different developmental stages of bamboo shoot buds was used to preliminarily study the function of TCP transcription factors in Ma bamboo, providing a series of differentially expressed genes that could be involved in the growth and development of bamboo shoots. Subsequently, the conservative function of *DlTCP12-C*, which negatively regulates axillary bud development and lateral branch growth, was confirmed in overexpressed transgenic *Arabidopsis*. This comprehensive study of TCP transcription factors in Ma bamboo provides several candidate genes worthy of further analysis, including the regulatory mechanism of bamboo shoot bud growth and development.

## Data Availability Statement

The datasets presented in this study can be found in online repositories. The names of the repository/repositories and accession number(s) can be found below: National Center for Biotechnology Information (NCBI) BioProject database under Accession Number PRJNA792061.

## Author Contributions

GQ and RZ conceived this project. KJ and YW designed experiments, interpreted the results, and wrote the manuscript. JX, ZL, HF, and BH performed the experiments and analyzed the data. YW provided technical guidance for the experiment. All authors read and approved the submission of this manuscript.

## Funding

This research was funded by the Zhejiang Science and Technology Major Program on Agricultural New Variety Breeding, Grant Number 2021C02070-4.

## Conflict of Interest

The authors declare that the research was conducted in the absence of any commercial or financial relationships that could be construed as a potential conflict of interest.

## Publisher's Note

All claims expressed in this article are solely those of the authors and do not necessarily represent those of their affiliated organizations, or those of the publisher, the editors and the reviewers. Any product that may be evaluated in this article, or claim that may be made by its manufacturer, is not guaranteed or endorsed by the publisher.

## Supplementary Material

Supplementary Figure S1 Multiple sequence alignment of TCP proteins in Ma bamboo.

Supplementary Figure S2 Distribution of Ka and Ks from paralogous (*Dl-Dl*) and orthologous (*Dl-Os, Dl-Pe*) gene pairs.

Supplementary Figure S3 GO annotation results of all differential genes and TCP members in the transcriptome.

Supplementary Figure S4 The results of transgene identification and expression detection.

Supplementary Table S1 The detailed information of TCP members in rice, *Arabidopsis thaliana*, and Moso bamboo.

Supplementary Table S2 List of primer sequences used in this study.

Supplementary Table S3 The detailed information of putative TCP members in Ma bamboo.

Supplementary Table S4 The detailed information of conserved motifs in DlTCPs.

Supplementary Table S5 Putative microRNA319-targeted binding sites of DlTCPs.

Supplementary Table S6 The detailed information and Ka/Ks ratio of putative orthologous and paralogous pairs.

Supplementary Table S7 The detailed information of three kinds of differentially expressed genes.
