# Supplementary material for: TCP Transcription Factors Involved in Shoot Development of Ma Bamboo (Dendrocalamus latiflorus Munro)
Source: Front Plant Sci. 2022 May 10;13:884443. doi: 10.3389/fpls.2022.884443 (PMC9127963; doi:10.3389/fpls.2022.884443)
Supplement: Supplementary Figure S1 — Multiple sequence alignment of TCP proteins in Ma bamboo. [file Data_Sheet_1.ZIP › Supplementary materials/Table S1 The detail information of TCP members in rice, Arabidopsis thaliana and moso bamboo.docx]

**Table S1** **|** The detail information of TCP members in rice*, Arabidopsis* and moso bamboo

| **Gene Name** | **Gene ID** | **Gene Name** | **Gene ID** | **Gene Name** | **Gene ID** |
| --- | --- | --- | --- | --- | --- |
| *OsTCP1* | LOC_Os01g11550 | *AtTCP1* | At1g67260 | *PeTCP1* | PH01000001G3420 |
| *OsTCP2* | LOC_Os01g55750 | *AtTCP2* | At4g18390 | *PeTCP2* | PH01000018G1460 |
| *OsTCP3* | LOC_Os01g69980 | *AtTCP3* | At1g53230 | *PeTCP3* | PH01000028G2500 |
| *OsTCP4* | LOC_Os02g42380 | *AtTCP4* | At3g15030 | *PeTCP4* | PH01000034G2110 |
| *OsTCP5* | LOC_Os02g51280 | *AtTCP5* | At5g60970 | *PeTCP5* | PH01000065G1920 |
| *OsTCP6* | LOC_Os02g51310 | *AtTCP6* | At5g41030 | *PeTCP6* | PH01000099G0140 |
| *OsTB1* | LOC_Os03g49880 | *AtTCP7* | At5g23280 | *PeTCP7* | PH01000131G1060 |
| *OsTCP8* | LOC_Os03g57190 | *AtTCP8* | At1g58100 | *PeTCP8* | PH01000135G0620 |
| *PCF1* | LOC_Os04g11830 | *AtTCP9* | At2g45680 | *PeTCP9* | PH01000155G0570 |
| *OsTCP10* | LOC_Os04g44440 | *AtTCP10* | At2g31070 | *PeTCP10* | PH01000256G0700 |
| *OsTCP11* | LOC_Os05g43760 | *AtTCP11* | At2g37000 | *PeTCP11* | PH01000423G0070 |
| *OsTCP12* | LOC_Os06g12230 | *AtTCP12* | At1g68800 | *PeTCP12* | PH01000519G0700 |
| *OsTCP13* | LOC_Os07g04510 | *AtTCP13* | At3g02150 | *PeTCP13* | PH01000602G0590 |
| *OsTCP14* | LOC_Os07g05720 | *AtTCP14* | At3g47620 | *PeTCP14* | PH01000767G0040 |
| *OsTCP15* | LOC_Os08g33530 | *AtTCP15* | At1g69690 | *PeTCP15* | PH01001418G0330 |
| *PCF2* | LOC_Os08g43160 | *AtTCP16* | At3g45150 | *PeTCP16* | PH01001480G0290 |
| *OsTCP17* | LOC_Os09g24480 | *AtTCP17* | At5g08070 |  |  |
| *OsTCP18* | LOC_Os09g34950 | *AtTCP18* | At3g18550 |  |  |
| *OsTCP19* | LOC_Os11g07460 | *AtTCP19* | At5g51910 |  |  |
| *OsTCP20* | LOC_Os12g02090 | *AtTCP20* | At3g27010 |  |  |
| *OsTCP21* | LOC_Os12g07480 | *AtTCP21* | At5g08330 |  |  |
| *OsTCP22* | LOC_Os12g42190 | *AtTCP22* | At1g72010 |  |  |
|  |  | *AtTCP23* | At1g35560 |  |  |
|  |  | *AtTCP24* | At1g30210 |  |  |
