# Supplementary material for: TCP Transcription Factors Involved in Shoot Development of Ma Bamboo (Dendrocalamus latiflorus Munro)
Source: Front Plant Sci. 2022 May 10;13:884443. doi: 10.3389/fpls.2022.884443 (PMC9127963; doi:10.3389/fpls.2022.884443)
Supplement: Supplementary Figure S1 — Multiple sequence alignment of TCP proteins in Ma bamboo. [file Data_Sheet_1.ZIP › Supplementary materials/Table S2 List of primer sequences used in this study.docx]

**Table S2** | List of primer sequences used in this study

| **Gene name** | **Forward primer sequences (5'-3')** | **Reverse primer sequences (5'-3')** | **Used** |
| --- | --- | --- | --- |
| DlTCP4-A | TCCTGAAAGGCTATTCTGGTGG | GGCTTGGCTTTGGCGAGT | qRT-PCR |
| DlTCP5-C | CGGCACAGCAAAGTGAACGG | GTGGCTGCGAGGATGGAAGG | qRT-PCR |
| DlTCP6-C | CGTCGTCATCGTCGCCATCC | CCCTCCCTGCGTGTTGTTGC | qRT-PCR |
| DlTCP7-B | GTACACGCAGAGCCAAATGCC | GCGGGAACGTCCAAATCGA | qRT-PCR |
| DlTCP9-A | CGAGGCAAGCGACGTGAT | AACCGTGAGTTGGCGAATA | qRT-PCR |
| DlTCP10-B | GTATGTCGCTGGGCAGATTG | CGGCAGTGATTACTCCCTTGA | qRT-PCR |
| DlTCP11-C | AAGCATCAGCAGGTCAGCAAGT | CCCGTGAGCAGCTCTGTGAA | qRT-PCR |
| DlTCP12-A | GGCTCAAGTCCGACGGCCAAAC | TGCAGCGCCTCCCAGAACGA | qRT-PCR |
| DlTCP13-B | CACAAGACGGACGGCGAGAC | TGGAGGAGCGGAGGGAGATG | qRT-PCR |
| DlTCP14-A | CTCGACCAAGGACCGGCACA | GGAGGAGCGGAGGGAGATGTT | qRT-PCR |
| DlTCP15-B | CGTGGTCGATGGCGAAGAGT | GGCGATGACGGGATAGTAGGC | qRT-PCR |
| DlTCP15-C | CAAGACGGACGGCGAGACCA | GGAGGAGCGGAGGGAGATGTT | qRT-PCR |
| DlTCP18-A | GGCTGCTGGATCAGGGAAGG | GACGCCGACATGACGTGGTT | qRT-PCR |
| DlTCP20-A | ACGGCGAGACCATCGAGTGG | GGTGCTGGAGGTGGTGAAAGG | qRT-PCR |
| DlTCP20-B | CTCCTTTCGCCACCGATGAT | TGCTGTGCCTGTCTTTCCTG | qRT-PCR |
| DlTCP23-C | AGCACGAGCACCAACAGCAGC | CGAGGGAGCCCATGAACGAC | qRT-PCR |
| GAPDH | CTCTTCGGCCAGAAGCCAGTCAC | TTGGCACCACCCTTCAAGTGAGC | qRT-PCR(internal reference) |
| DlTCP5-C(pMDC43) | atgaactatacaaaggcgcgccaATGGCCTCCCGGGACGTTG | ccgctctagaactagttaatTCACCGGGACTCCTCCTC | Subcellular localization |
| DlTCP7-B(pMDC43) | atgaactatacaaaggcgcgccaATGGACGTCGCCGGAGAT | ccgctctagaactagttaatCTATGAGTCGCTGGTGCTCA | Subcellular localization |
| DlTCP9-A(pMDC43) | atgaactatacaaaggcgcgccaATGGACATACCGCTTTACCA | ccgctctagaactagttaatTCAGTAGAACCGTGAGTTGGC | Subcellular localization |
| DlTCP23-C(pMDC43) | atgaactatacaaaggcgcgccaATGGACCAGGGAGCGGC | ccgctctagaactagttaatTTACTGTGGGCTTGCGGC | Subcellular localization |
| DlTCP5-C(pGBKT7) | tggccatggaggccgaattcATGGCCTCCCGGGACGTTG | cgctgcaggtcgacggatccTCACCGGGACTCCTCCTC | transactivation activity |
| DlTCP7-B(pGBKT7) | tggccatggaggccgaattcATGGACGTCGCCGGAGAT | cgctgcaggtcgacggatccCTATGAGTCGCTGGTGCTCA | transactivation activity |
| DlTCP9-A(pGBKT7) | tggccatggaggccgaattcATGGACATACCGCTTTACCA | cgctgcaggtcgacggatccTCAGTAGAACCGTGAGTTGGC | transactivation activity |
| DlTCP23-C(pGBKT7) | tggccatggaggccgaattcATGGACCAGGGAGCGGC | cgctgcaggtcgacggatccTTACTGTGGGCTTGCGGC | transactivation activity |
| DlTCP12-C(pCAMBIA1300) | agaacacgggggacgagctcATGTTTCCTTTCTGTGATTCC | tagtccattctagaggatccGTAGAACCGTGAGTTGGGA | transgenic *Arabidopsis* |
