# Supplementary material for: TCP Transcription Factors Involved in Shoot Development of Ma Bamboo (Dendrocalamus latiflorus Munro)
Source: Front Plant Sci. 2022 May 10;13:884443. doi: 10.3389/fpls.2022.884443 (PMC9127963; doi:10.3389/fpls.2022.884443)
Supplement: Supplementary Figure S1 — Multiple sequence alignment of TCP proteins in Ma bamboo. [file Data_Sheet_1.ZIP › Supplementary materials/Table S3 The detail information of putative TCP members in ma bamboo.docx]

**Table S3** **|** The detail information of putative TCP members in ma bamboo

| **Gene Name** | **Gene ID** | **Chr** | **start** | **end** | **strand** | **AA** | **PI** | **MW** | **Subcellular Location** |
| --- | --- | --- | --- | --- | --- | --- | --- | --- | --- |
| DlTCP1-A | evm.model.FRAGSCAFF_390.234 | chr1.1 | 8316574 | 8320160 | - | 566 | 6.43 | 59339.82 | Nuclear |
| DlTCP2-A | evm.model.FRAGSCAFF_223.793 | chr1.1 | 49319383 | 49323000 | + | 404 | 6.86 | 43580.09 | Nuclear |
| DlTCP3-A | evm.model.FRAGSCAFF_223.865 | chr1.1 | 50030829 | 50032916 | - | 278 | 9.39 | 30135.83 | Nuclear |
| DlTCP4-A | evm.model.FRAGSCAFF_346.252 | chr4.1 | 18064649 | 18069905 | - | 525 | 10.59 | 55225.91 | Nuclear |
| DlTCP5-A | evm.model.FRAGSCAFF_250.230 | chr4.1 | 35333775 | 35334822 | + | 208 | 10.39 | 21260.03 | Nuclear |
| DlTCP6-A | evm.model.ORIGINAL_2067.70 | chr4.1 | 43711101 | 43713294 | + | 391 | 9.22 | 39229.31 | Nuclear |
| DlTCP7-A | evm.model.FRAGSCAFF_245.134 | chr4.1 | 48339348 | 48339959 | - | 204 | 9.87 | 20976.62 | Nuclear |
| DlTCP8-A | evm.model.FRAGSCAFF_230.919 | chr7.1 | 9887920 | 9889047 | + | 367 | 7.14 | 38226.44 | Chloroplast, Nuclear |
| DlTCP9-A | evm.model.FRAGSCAFF_158.468 | chr7.1 | 45309852 | 45311040 | + | 341 | 8.32 | 37247.86 | Nuclear |
| DlTCP10-A | evm.model.FRAGSCAFF_411.81 | chr7.1 | 51000725 | 51005119 | + | 384 | 9.03 | 39756.97 | Nuclear |
| DlTCP11-A | evm.model.ORIGINAL_2393.5 | chr10.1 | 9278148 | 9278992 | - | 170 | 6.84 | 17637.86 | Nuclear |
| DlTCP12-A | evm.model.FRAGSCAFF_19.1664 | chr10.1 | 42803888 | 42804896 | + | 183 | 10.11 | 19422.03 | Nuclear |
| DlTCP13-A | evm.model.FRAGSCAFF_426.489 | chr13.1 | 30246645 | 30248618 | + | 280 | 6.97 | 29653.02 | Nuclear |
| DlTCP14-A | evm.model.ORIGINAL_2555.48 | chr16.1 | 8118241 | 8119958 | + | 379 | 9.54 | 38854.07 | Nuclear |
| DlTCP15-A | evm.model.ORIGINAL_4560.55 | chr19.1 | 3209343 | 3216597 | - | 438 | 9.4 | 46226.37 | Nuclear |
| DlTCP16-A | evm.model.FRAGSCAFF_356.195 | chr23.1 | 31875175 | 31877322 | + | 267 | 5.37 | 29214.52 | Nuclear |
| DlTCP17-A | evm.model.FRAGSCAFF_455.133 | chr23.1 | 41963841 | 41965703 | - | 373 | 5.62 | 38304.79 | Nuclear |
| DlTCP18-A | evm.model.ORIGINAL_5142.133 | chr26.1 | 28223637 | 28224801 | + | 240 | 7.1 | 26327.4 | Nuclear |
| DlTCP19-A | evm.model.FRAGSCAFF_171.308 | chr26.1 | 36995789 | 36996931 | - | 340 | 5.48 | 34911.91 | Nuclear |
| DlTCP20-A | evm.model.FRAGSCAFF_43.109 | chr32.1 | 3028258 | 3030092 | - | 369 | 7.37 | 38297.52 | Nuclear |
| DlTCP21-A | evm.model.FRAGSCAFF_113.191 | chr35.1 | 3697571 | 3699397 | - | 399 | 6.07 | 41015.81 | Nuclear |
| DlTCP22-A | evm.model.FRAGSCAFF_149.495 | chr35.1 | 23604274 | 23605161 | + | 296 | 6.17 | 31239.88 | Nuclear |
| DlTCP1-B | evm.model.FRAGSCAFF_196.205 | chr2.1 | 6937488 | 6940100 | - | 450 | 6.5 | 46916.15 | Nuclear |
| DlTCP2-B | evm.model.FRAGSCAFF_51.282 | chr2.1 | 48544585 | 48546514 | + | 324 | 6.14 | 34296.69 | Nuclear |
| DlTCP3-B | evm.model.FRAGSCAFF_51.339 | chr2.1 | 49276357 | 49279067 | - | 270 | 8.09 | 29349.94 | Nuclear |
| DlTCP4-B | evm.model.ORIGINAL_2711.103 | chr2.1 | 61167804 | 61169488 | + | 324 | 6.53 | 33616.1 | Nuclear |
| DlTCP5-B | evm.model.FRAGSCAFF_68.184 | chr5.1 | 35778273 | 35781070 | + | 252 | 6.96 | 26473.9 | Nuclear |
| DlTCP6-B | evm.model.FRAGSCAFF_68.185 | chr5.1 | 35799589 | 35800554 | + | 210 | 9.82 | 21571.34 | Nuclear |
| DlTCP7-B | evm.model.FRAGSCAFF_68.907 | chr5.1 | 43439620 | 43441922 | - | 397 | 9.09 | 39678.76 | Nuclear |
| DlTCP8-B | evm.model.ORIGINAL_1671.46 | chr5.1 | 48437895 | 48439777 | - | 235 | 10.09 | 23590.6 | Nuclear |
| DlTCP9-B | evm.model.FRAGSCAFF_387.397 | chr8.1 | 47851482 | 47852926 | + | 353 | 8.78 | 38241.15 | Nuclear |
| DlTCP10-B | evm.model.FRAGSCAFF_387.1791 | chr8.1 | 53801073 | 53820691 | + | 768 | 9.22 | 81796.66 | Peroxisome |
| DlTCP11-B | evm.model.FRAGSCAFF_371.561 | chr11.1 | 27743483 | 27744593 | + | 193 | 10.59 | 20698.61 | Nuclear |
| DlTCP12-B | evm.model.FRAGSCAFF_133.438 | chr14.1 | 30848141 | 30850387 | + | 285 | 6.92 | 30124.78 | Nuclear |
| DlTCP13-B | evm.model.FRAGSCAFF_259.279 | chr17.1 | 7772402 | 7774229 | + | 379 | 7.17 | 38658.91 | Cell membrane, Nuclear |
| DlTCP14-B | evm.model.ORIGINAL_1117.20 | chr20.1 | 3169806 | 3174814 | + | 439 | 9.42 | 46048.14 | Nuclear |
| DlTCP15-B | evm.model.ORIGINAL_5293.136 | chr24.1 | 31667598 | 31669074 | - | 363 | 5.95 | 37508.93 | Nuclear |
| DlTCP16-B | evm.model.FRAGSCAFF_62.220 | chr27.1 | 17279674 | 17280369 | - | 232 | 5.57 | 25286.85 | Nuclear |
| DlTCP17-B | evm.model.FRAGSCAFF_425.311 | chr33.1 | 4577358 | 4580090 | - | 367 | 8.85 | 38116.38 | Nuclear |
| DlTCP18-B | evm.model.FRAGSCAFF_102.2 | chr36.1 | 4483629 | 4485442 | - | 414 | 6.2 | 42271.17 | Nuclear |
| DlTCP19-B | evm.model.ORIGINAL_4625.58 | chr36.1 | 23313626 | 23316712 | + | 301 | 6.66 | 31624.34 | Nuclear |
| DlTCP20-B | evm.model.FRAGSCAFF_16.82 | chr36.1 | 24255993 | 24256853 | + | 287 | 9.86 | 32050.43 | Nuclear |
| DlTCP1-C | evm.model.FRAGSCAFF_173.278 | chr3.1 | 25993322 | 25995871 | + | 487 | 6.48 | 52062.46 | Nuclear |
| DlTCP2-C | evm.model.FRAGSCAFF_173.322 | chr3.1 | 26491737 | 26494032 | - | 270 | 7.25 | 28994.37 | Nuclear |
| DlTCP3-C | evm.model.FRAGSCAFF_50.64 | chr3.1 | 33686583 | 33689115 | - | 443 | 6.34 | 46175.41 | Nuclear |
| DlTCP4-C | evm.model.FRAGSCAFF_50.482 | chr3.1 | 38028737 | 38032238 | - | 328 | 6.47 | 34149.49 | Nuclear |
| DlTCP5-C | evm.model.ORIGINAL_5780.219 | chr6.1 | 27433052 | 27434237 | + | 208 | 10.08 | 21297.95 | Nuclear |
| DlTCP6-C | evm.model.FRAGSCAFF_263.186 | chr6.1 | 34062900 | 34065075 | + | 391 | 9.12 | 39026.09 | Nuclear |
| DlTCP7-C | evm.model.ORIGINAL_563.270 | chr6.1 | 38383089 | 38384673 | - | 207 | 9.87 | 21107.69 | Nuclear |
| DlTCP8-C | evm.model.FRAGSCAFF_125.27 | chr9.1 | 40539016 | 40540035 | + | 273 | 9.47 | 30473.41 | Nuclear |
| DlTCP9-C | evm.model.FRAGSCAFF_190.1395 | chr12.1 | 31835011 | 31835856 | + | 197 | 10.6 | 20746.66 | Nuclear |
| DlTCP10-C | evm.model.ORIGINAL_4683.120 | chr15.1 | 24843919 | 24847028 | + | 280 | 9.14 | 29524.08 | Nuclear |
| DlTCP11-C | evm.model.ORIGINAL_2012.42 | chr18.1 | 3562929 | 3569055 | - | 387 | 9.03 | 40436.84 | Nuclear |
| DlTCP12-C | evm.model.FRAGSCAFF_292.158 | chr18.1 | 7448246 | 7449404 | - | 350 | 8.5 | 38227.03 | Nuclear |
| DlTCP13-C | evm.model.FRAGSCAFF_292.55 | chr18.1 | 8535252 | 8536649 | - | 128 | 10.09 | 13510.46 | Nuclear |
| DlTCP14-C | evm.model.FRAGSCAFF_400.785 | chr18.1 | 10711976 | 10712893 | - | 306 | 6.76 | 32223.04 | Nuclear |
| DlTCP15-C | evm.model.FRAGSCAFF_20.238 | chr18.1 | 20269483 | 20270815 | + | 384 | 8.61 | 38988.92 | Nuclear |
| DlTCP16-C | evm.model.ORIGINAL_1651.31 | chr18.1 | 33883895 | 33885787 | - | 455 | 9.5 | 48794.83 | Nuclear |
| DlTCP17-C | evm.model.ORIGINAL_979.393 | chr22.1 | 16656471 | 16659515 | - | 437 | 8.84 | 45939.98 | Nuclear |
| DlTCP18-C | evm.model.FRAGSCAFF_120.35 | chr25.1 | 15590200 | 15590994 | + | 265 | 6.26 | 29177.53 | Nuclear |
| DlTCP19-C | evm.model.FRAGSCAFF_203.342 | chr25.1 | 23229312 | 23231744 | - | 364 | 5.53 | 37622.12 | Nuclear |
| DlTCP20-C | evm.model.FRAGSCAFF_391.125 | chr28.1 | 16080589 | 16081245 | + | 219 | 6.71 | 23843.6 | Nuclear |
| DlTCP21-C | evm.model.ORIGINAL_5941.63 | chr28.1 | 18691154 | 18691634 | + | 135 | 5.7 | 14648.25 | Chloroplast, Nuclear |
| DlTCP22-C | evm.model.ORIGINAL_3472.93 | chr28.1 | 23269189 | 23270469 | - | 337 | 5.16 | 34319.02 | Nuclear |
| DlTCP23-C | evm.model.FRAGSCAFF_294.199 | chr34.1 | 8394054 | 8395935 | - | 363 | 7.97 | 37394.56 | Nuclear |
| DlTCP24-C | evm.model.ORIGINAL_5718.428 | chr34.1 | 24228754 | 24230694 | + | 406 | 6.15 | 41725.7 | Nuclear |
