# Supplementary material for: TCP Transcription Factors Involved in Shoot Development of Ma Bamboo (Dendrocalamus latiflorus Munro)
Source: Front Plant Sci. 2022 May 10;13:884443. doi: 10.3389/fpls.2022.884443 (PMC9127963; doi:10.3389/fpls.2022.884443)
Supplement: Supplementary Figure S1 — Multiple sequence alignment of TCP proteins in Ma bamboo. [file Data_Sheet_1.ZIP › Supplementary materials/Table S4 The detail information of conserved motifs in DlTCPs.docx]

**Table S4** **|** The detail information of conserved motifs in DlTCPs

| **Motif** | **Length** | **Protein Sequence** | **Pfam Domain** |
| --- | --- | --- | --- |
| 1 | 50 | DRHTKVDGRGRRIRMPALCAARVFQLTRELGHKSDGETIEWLLQQAEPAI | TCP |
| 2 | 15 | IAATGTGTIPANFTS |  |
| 3 | 13 | RVSRVFGGKDRHS |  |
| 4 | 50 | SSPSPLLLNFHSGSVGLDVQPSPSVAAAAADLSRKRRWEQEMQQQQQQQQ |  |
| 5 | 49 | VGGHIGFAPMFTGHAAAAMPGLELGLSQDGHIGVLTAQSLSQFYHQVGG |  |
| 6 | 29 | VGGSGGGGGEGHMGILAALNAYRTQAATD |  |
| 7 | 29 | QQLTKSGCSSTSETSKGSVLSLSRSESRV |  |
| 8 | 35 | MDVAGDAGGGRRPNFPLQLLEKKEEQPCSSSAAGG |  |
| 9 | 21 | SVPSGLHFMNFPAPMALLPGQ |  |
| 10 | 22 | MAGYTQSQMPGTVWMVPSNNTQ |  |
