# Supplementary material for: TCP Transcription Factors Involved in Shoot Development of Ma Bamboo (Dendrocalamus latiflorus Munro)
Source: Front Plant Sci. 2022 May 10;13:884443. doi: 10.3389/fpls.2022.884443 (PMC9127963; doi:10.3389/fpls.2022.884443)
Supplement: Supplementary Figure S1 — Multiple sequence alignment of TCP proteins in Ma bamboo. [file Data_Sheet_1.ZIP › Supplementary materials/Table S5 Putative microRNA319-targeted binding sites of the DlTCPs.docx]

**Table S5** **|** Putative microRNA319-targeted binding sites of the DlTCPs

| miRNA Name | Transcript name | Position | Folding | Heteroduplex | p value |
| --- | --- | --- | --- | --- | --- |
| dla-miR319 | DlTCP3-C | 338 | -12.00 | TTGCTTCAAGAATTTGATCACT \|\|:\|:\|\| \|\|:: \|\|\|\|\| AATGGAG-GGCGAAGTAAGTGA | 3.1E-1 |
| dla-miR319 | DlTCP2-B | 212 | -14.90 | TTTCCTCCC--TTTATTCTCG  \|\|\|\|\|\| \|\|:\|\|\|\| \|  AATGGAGGGCGAAGTAAGTGA | 2.69E-1 |
| dla-miR319 | DlTCP12-B | 350 | -15.60 | GATTCTCCCCCTTT-TTTACT  :\|\|\|\|\| \|\|\|: \|\|:\|\|\| AATGGAGGGCGAAGTAAGTGA | 1.32E-1 |
| dla-miR319 | DlTCP15-A | 2679 | -13.80 | TTGCC-CTTGAAAAGTTTCACT \|\|:\|\| \|::\| \|\|\|\|\|\| AATGGAGGGC-GAAGTAAGTGA | 1.27E-1 |
| dla-miR319 | DlTCP14-B | 2422 | -13.30 | CTTGGTGCCATTTCATTCACC  \| \|\| :\|\|\|\|\|\|\|\|\|  AATGGAGGGCGAAGTAAGTGA | 1.61E-1 |
