# Supplementary material for: TCP Transcription Factors Involved in Shoot Development of Ma Bamboo (Dendrocalamus latiflorus Munro)
Source: Front Plant Sci. 2022 May 10;13:884443. doi: 10.3389/fpls.2022.884443 (PMC9127963; doi:10.3389/fpls.2022.884443)
Supplement: Supplementary Figure S1 — Multiple sequence alignment of TCP proteins in Ma bamboo. [file Data_Sheet_1.ZIP › Supplementary materials/Table S6 The detail information of putative orthologous and paralogous pairs.docx]

**Table S4.1** **|** Orthologous (*Dl-At, Dl-Os, Dl-Pe*) and paralogous (*Dl-Dl*) of TCP gene pairs

| ***Dl-Os*** | ***Dl-Pe*** | ***Dl-Dl*** |
| --- | --- | --- |
| *DlTCP6-C/OsTCP5* | *DlTCP19-A/PeTCP7* | *DlTCP17-B/DlTCP23-C* |
| *DlTCP11-A/PCF1* | *DlTCP12-A/PeTCP3* | *DlTCP21-A/DlTCP18-B* |
| *DlTCP1-B/* *OsTCP1* | *DlTCP11-B/PeTCP1* | *DlTCP8-A/DlTCP16-C* |
|  | *DlTCP8-C/PeTCP13* | *DlTCP17-A/DlTCP15-B* |
|  | *DlTCP11-C/PeTCP14* | *DlTCP8-B/DlTCP7-C* |
|  |  | *DlTCP6-B/DlTCP5-C* |
|  |  | *DlTCP9-B/DlTCP12-C* |
|  |  | *DlTCP16-A/DlTCP18-C* |
|  |  | *DlTCP18-A/DlTCP16-B* |
|  |  | *DlTCP3-A/DlTCP3-B* |
|  |  | *DlTCP13-A/DlTCP12-B* |
|  |  | *DlTCP2-B/DlTCP1-C* |
|  |  | *DlTCP19-B/DlTCP14-C* |
|  |  | *DlTCP14-B/DlTCP17-C* |

**Table S4.2** **|** The Ka and Ks value of orthologous and paralogous gene pairs

| **Seq_1** | **Seq_2** | **Ka** | **Ks** | **Ka/Ks** |
| --- | --- | --- | --- | --- |
| *DlTCP6-C* | *OsTCP5* | 0.0404556 | 0.460558 | 0.08784 |
| *DlTCP11-A* | *PCF1* | 0.1153238 | 0.251992 | 0.45765 |
| *DlTCP1-B* | *OsTCP1* | 0.0732137 | 0.308378 | 0.237415 |
| *DlTCP19-A* | *PeTCP7* | 0.0733944 | 0.113881 | 0.644484 |
| *DlTCP12-A* | *PeTCP3* | 0.0638825 | 0.219142 | 0.291513 |
| *DlTCP11-B* | *PeTCP1* | 0.0520655 | 0.13625 | 0.382134 |
| *DlTCP8-C* | *PeTCP13* | 0.0344605 | 0.111424 | 0.309274 |
| *DlTCP11-C* | *PeTCP14* | 0.0551885 | 0.076548 | 0.720963 |
| *DlTCP17-B* | *DlTCP23-C* | 0.0382248 | 0.227619 | 0.167934 |
| *DlTCP21-A* | *DlTCP18-B* | 0.0286869 | 0.186462 | 0.153849 |
| *DlTCP8-A* | *DlTCP16-C* | 0.1394553 | 0.187671 | 0.743086 |
| *DlTCP17-A* | *DlTCP15-B* | 0.0347882 | 0.170517 | 0.204016 |
| *DlTCP8-B* | *DlTCP7-C* | 0.0345418 | 0.165427 | 0.208804 |
| *DlTCP6-B* | *DlTCP5-C* | 0.0418687 | 0.172809 | 0.242282 |
| *DlTCP9-B* | *DlTCP12-C* | 0.0453702 | 0.076991 | 0.589295 |
| *DlTCP16-A* | *DlTCP18-C* | 0.0848439 | 0.22622 | 0.375051 |
| *DlTCP18-A* | *DlTCP16-B* | 0.07844 | 0.166406 | 0.471378 |
| *DlTCP3-A* | *DlTCP3-B* | 0.0652015 | 0.108813 | 0.599208 |
| *DlTCP13-A* | *DlTCP12-B* | 0.0447091 | 0.13826 | 0.323369 |
| *DlTCP2-B* | *DlTCP1-C* | 0.04458 | 0.133122 | 0.334881 |
| *DlTCP19-B* | *DlTCP14-C* | 0.04912 | 0.169593 | 0.289634 |
| *DlTCP14-B* | *DlTCP17-C* | 0.042092 | 0.147432 | 0.2855 |
