# Supplementary material for: TCP Transcription Factors Involved in Shoot Development of Ma Bamboo (Dendrocalamus latiflorus Munro)
Source: Front Plant Sci. 2022 May 10;13:884443. doi: 10.3389/fpls.2022.884443 (PMC9127963; doi:10.3389/fpls.2022.884443)
Supplement: Supplementary Figure S1 — Multiple sequence alignment of TCP proteins in Ma bamboo. [file Data_Sheet_1.ZIP › Supplementary materials/Table S7 The detailed information of three kinds of differentially expressed genes.docx]

**Table S7** **|** The detailed information of three kinds of differentially expressed genes

| **Gene Name** | **Gene ID** |
| --- | --- |
| Auxin response factor 2 | evm.TU.FRAGSCAFF_113.28 |
| Auxin response factor 1 | evm.TU.FRAGSCAFF_119.155 |
| Auxin responsive protein SAUR71 | evm.TU.FRAGSCAFF_120.182 |
| Auxin response factor 21 | evm.TU.FRAGSCAFF_120.494 |
| Auxin response factor 9 | evm.TU.FRAGSCAFF_121.145 |
| Auxin responsive protein | evm.TU.FRAGSCAFF_125.139 |
| Auxin responsive protein IAA15 | evm.TU.FRAGSCAFF_128.286 |
| Auxin response factor 4 | evm.TU.FRAGSCAFF_129.439 |
| Gibberellin regulated protein8 | evm.TU.FRAGSCAFF_121.301 |
| Gibberellin regulated protein1 | evm.TU.FRAGSCAFF_123.186 |
| Gibberellin receptor GID1 | evm.TU.FRAGSCAFF_132.67 |
| Gibberellin regulated protein4 | evm.TU.FRAGSCAFF_245.491 |
| Gibberellin receptor GID2 | evm.TU.FRAGSCAFF_83.81 |
| Gibberellin regulated protein3 | evm.TU.ORIGINAL_668.55 |
| Gibberellin regulated protein9 | evm.TU.FRAGSCAFF_236.60 |
| Gibberellin regulated protein14 | evm.TU.FRAGSCAFF_330.199 |
| bZIP transcription factor 27 | evm.TU.FRAGSCAFF_171.418 |
| Abscisic acid insensitive 5 | evm.TU.FRAGSCAFF_173.535 |
| bZIP transcription factor ABI5 | evm.TU.FRAGSCAFF_173.823 |
| Ethylene responsive transcription factor ERF110 | evm.TU.FRAGSCAFF_173.885 |
| Abscisic acid receptor PYL3 | evm.TU.FRAGSCAFF_180.48 |
| Abscisic acid receptor PYL5 | evm.TU.FRAGSCAFF_184.116 |
| Serine/threonine-protein kinase SAPK1 | evm.TU.FRAGSCAFF_187.128 |
| Protein phosphatase 2C | evm.TU.FRAGSCAFF_188.320 |
| Ethylene-responsive transcription factor 4 | evm.TU.FRAGSCAFF_199.270 |
| Abscisic acid receptor PYL10 | evm.TU.FRAGSCAFF_216.123 |
| Abscisic acid insensitive 5-like protein 2 | evm.TU.FRAGSCAFF_236.700 |
| bZIP transcription factor | evm.TU.FRAGSCAFF_259.402 |
| bZIP transcription factor 23 | evm.TU.FRAGSCAFF_263.275 |
| bZIP transcription factor RISBZ1 | evm.TU.FRAGSCAFF_273.188 |
| Protein phosphatase 2C-1 | evm.TU.FRAGSCAFF_301.923 |
| Abscisic acid insensitive 5-like protein 1 | evm.TU.FRAGSCAFF_317.215 |
| Abscisic acid receptor PYL4 | evm.TU.FRAGSCAFF_377.40 |
| WRKY transcription factor 53 | evm.TU.FRAGSCAFF_426.154 |
| WRKY transcription factor 24 | evm.TU.FRAGSCAFF_132.282 |
| WRKY transcription factor 38 | evm.TU.FRAGSCAFF_129.1521 |
| WRKY transcription factor 3 | evm.TU.FRAGSCAFF_149.122 |
| WRKY transcription factor 50 | evm.TU.FRAGSCAFF_132.890 |
| WRKY transcription factor 52 | evm.TU.FRAGSCAFF_133.329 |
| WRKY transcription factor 4 | evm.TU.FRAGSCAFF_173.591 |
| WRKY transcription factor 12 | evm.TU.FRAGSCAFF_190.1273 |
| WRKY transcription factor 57 | evm.TU.FRAGSCAFF_236.101 |
| WRKY transcription factor 66 | evm.TU.FRAGSCAFF_232.334 |
| WRKY transcription factor 12 | evm.TU.FRAGSCAFF_250.128 |
| WRKY transcription factor 9 | evm.TU.FRAGSCAFF_263.300 |
| NAC transcription factor 48 | evm.TU.FRAGSCAFF_129.1078 |
| NAC transcription factor 82 | evm.TU.FRAGSCAFF_129.1102 |
| NAC transcription factor 90 | evm.TU.FRAGSCAFF_129.1234 |
| NAC transcription factor 2 | evm.TU.FRAGSCAFF_19.1948 |
| NAC transcription factor 35 | evm.TU.FRAGSCAFF_320.605 |
| NAC transcription factor 74 | evm.TU.FRAGSCAFF_390.552 |
| NAC transcription factor 3 | evm.TU.ORIGINAL_1671.83 |
| NAC transcription factor 53 | evm.TU.ORIGINAL_3630.48 |
| MYB transcription factor SRM1 | evm.TU.FRAGSCAFF_190.401 |
| MYB-like transcription factor | evm.TU.FRAGSCAFF_244.16 |
| Transcription repressor MYB4 | evm.TU.FRAGSCAFF_113.173 |
| MYB related protein | evm.TU.FRAGSCAFF_121.442 |
| MYB-like transcription factor | evm.TU.FRAGSCAFF_129.1589 |
| MYB-like transcription factor | evm.TU.FRAGSCAFF_133.304 |
| MYB transcription factor | evm.TU.FRAGSCAFF_149.319 |
| MYB transcription factor 3R-4 | evm.TU.FRAGSCAFF_167.16 |
| MYB transcription factor 17 | evm.TU.FRAGSCAFF_19.1613 |
| MYB transcription factor 41 | evm.TU.FRAGSCAFF_190.1345_evm.TU.FRAGSCAFF_190.1346 |
| MYB transcription factor 111 | evm.TU.FRAGSCAFF_203.151 |
| MYB transcription factor 73 | evm.TU.FRAGSCAFF_312.90 |
| MYB transcription factor 44 | evm.TU.FRAGSCAFF_364.186 |
| MYB transcription factor 36 | evm.TU.FRAGSCAFF_366.170 |
| MYB transcription factor 4 | evm.TU.FRAGSCAFF_371.615 |
| MYB transcription factor 3 | evm.TU.FRAGSCAFF_50.796 |
| Expansin A2 | evm.TU.FRAGSCAFF_129.1512 |
| Expansin C3 | evm.TU.FRAGSCAFF_173.601 |
| Expansin C6 | evm.TU.FRAGSCAFF_212.270 |
| Expansin B4 | evm.TU.FRAGSCAFF_216.214 |
| Expansin A4 | evm.TU.FRAGSCAFF_377.6 |
| Expansin A7 | evm.TU.FRAGSCAFF_387.1143 |
| Expansin A8 | evm.TU.FRAGSCAFF_411.279 |
| Expansin A5 | evm.TU.FRAGSCAFF_68.921 |
| Growth regulating factor 10 | evm.TU.FRAGSCAFF_244.50 |
| Growth interacting factor 1 | evm.TU.FRAGSCAFF_158.668 |
| Growth regulating factor 12 | evm.TU.FRAGSCAFF_19.1057 |
| Growth regulating factor 3 | evm.TU.FRAGSCAFF_212.177 |
| Growth regulating factor 10 | evm.TU.FRAGSCAFF_244.50 |
| Growth regulating factor 6 | evm.TU.FRAGSCAFF_292.297 |
| Growth regulating factor 4 | evm.TU.ORIGINAL_3711.137 |
| Growth regulating factor 2 | evm.TU.ORIGINAL_600.63 |
| Growth regulating factor 7 | evm.TU.ORIGINAL_6034.44 |
| Dof zinc finger protein 8 | evm.TU.FRAGSCAFF_123.260 |
| Dof zinc finger protein 7 | evm.TU.FRAGSCAFF_132.437 |
| Dof zinc finger protein 9 | evm.TU.FRAGSCAFF_149.384 |
| Dof zinc finger protein MNB1A | evm.TU.FRAGSCAFF_154.227 |
| Dof zinc finger protein 3 | evm.TU.FRAGSCAFF_158.856 |
| Dof zinc finger protein 1 | evm.TU.FRAGSCAFF_19.1009 |
| Dof zinc finger protein 4 | evm.TU.FRAGSCAFF_320.506 |
| Dof zinc finger protein 5 | evm.TU.FRAGSCAFF_349.78 |
| Dof zinc finger protein 2 | evm.TU.FRAGSCAFF_68.441 |
| Dof zinc finger protein 6 | evm.TU.ORIGINAL_3711.90 |
