# Supplementary figures and images for: TCP Transcription Factors Involved in Shoot Development of Ma Bamboo (Dendrocalamus latiflorus Munro)
Source: Front Plant Sci. 2022 May 10;13:884443. doi: 10.3389/fpls.2022.884443 (PMC9127963; doi:10.3389/fpls.2022.884443)

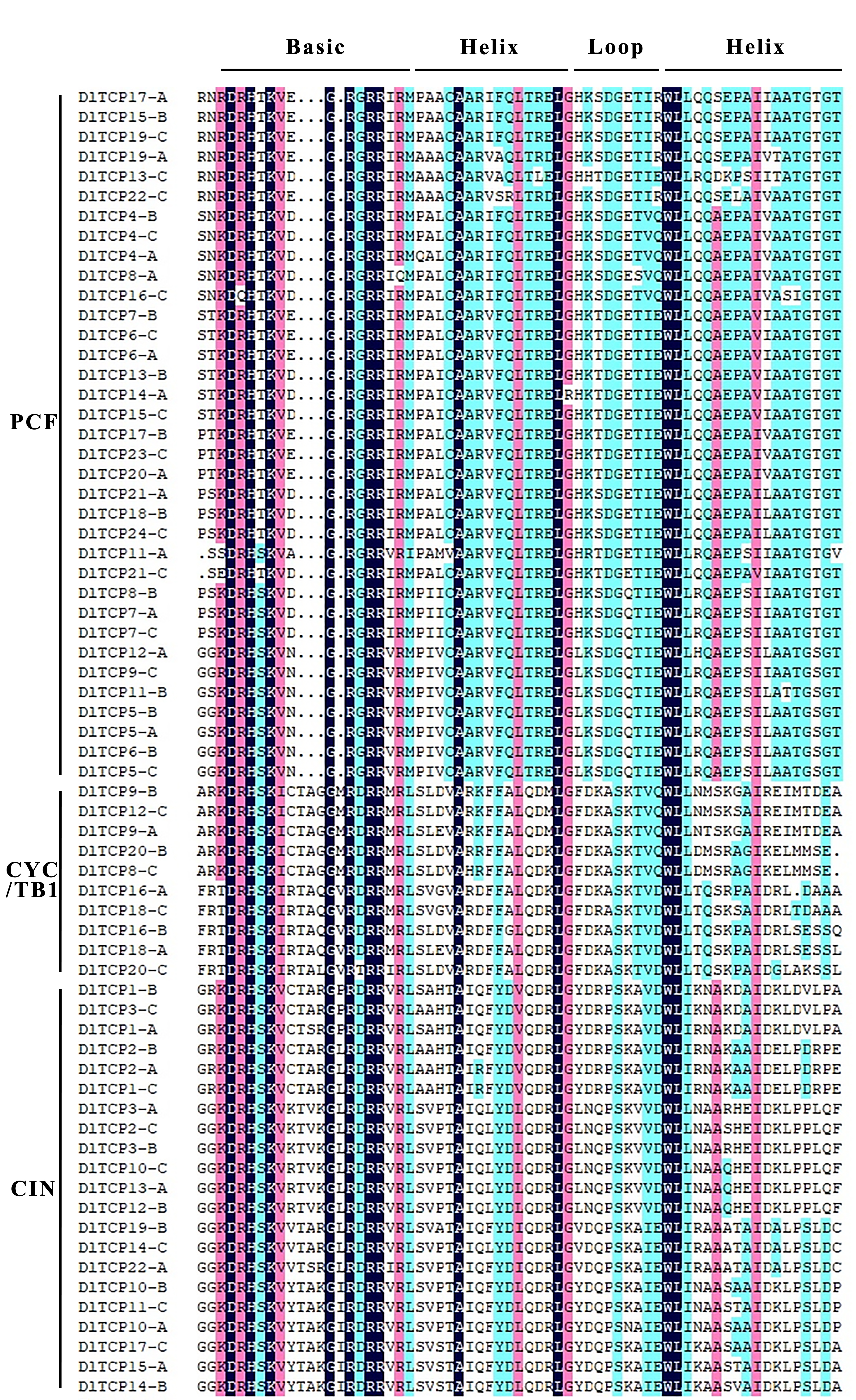

Supplement: Supplementary Figure S1 — Multiple sequence alignment of TCP proteins in Ma bamboo. [file Data_Sheet_1.ZIP › Supplementary materials/Figure S1 Multiple sequence alignment of TCP proteins in ma bamboo.jpg]

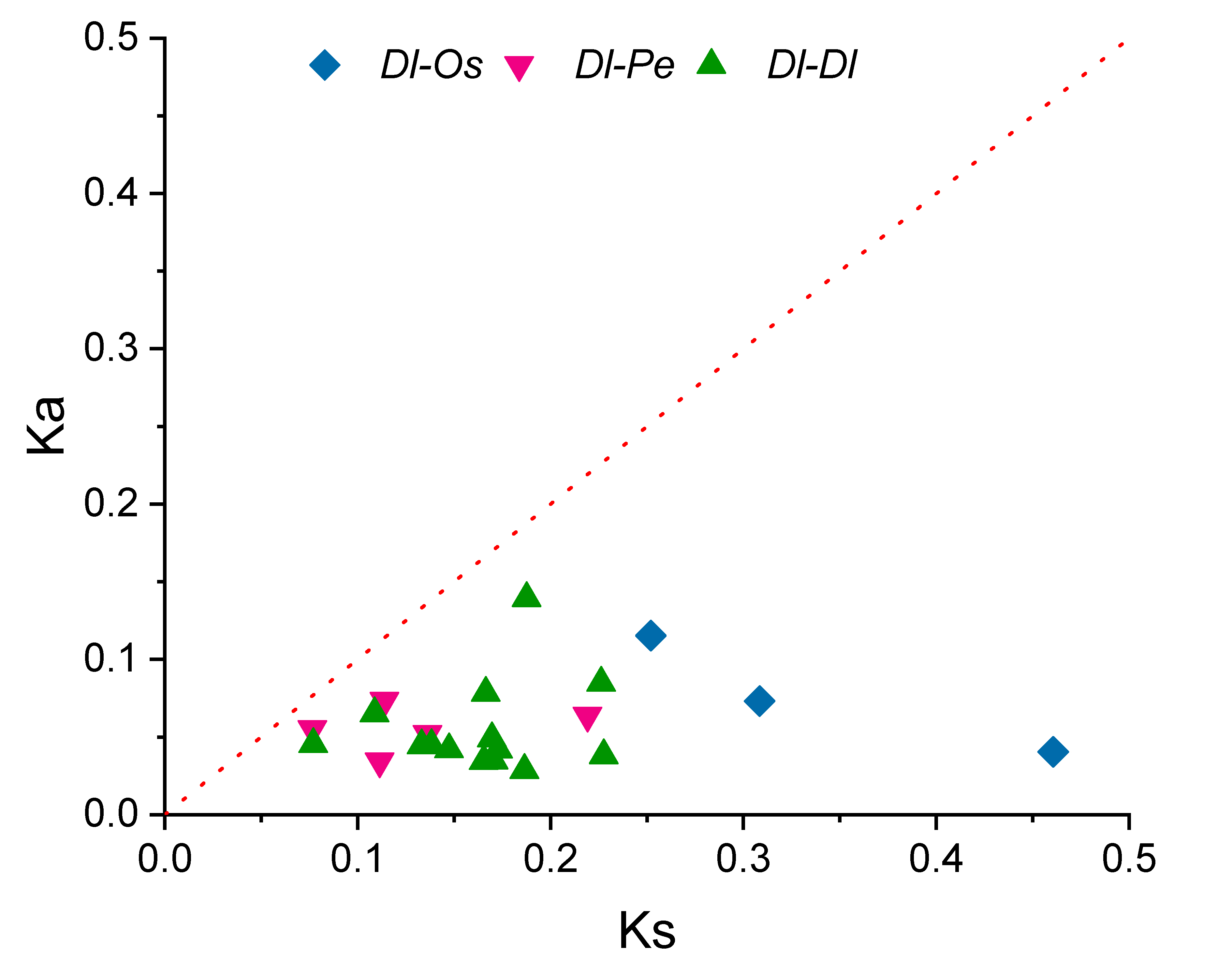

Supplement: Supplementary Figure S1 — Multiple sequence alignment of TCP proteins in Ma bamboo. [file Data_Sheet_1.ZIP › Supplementary materials/Figure S2 Distribution of Ka and Ks from paralogous (Dl-Dl) and orthologous (Dl-Os,Dl-Pe) gene pairs.jpg]

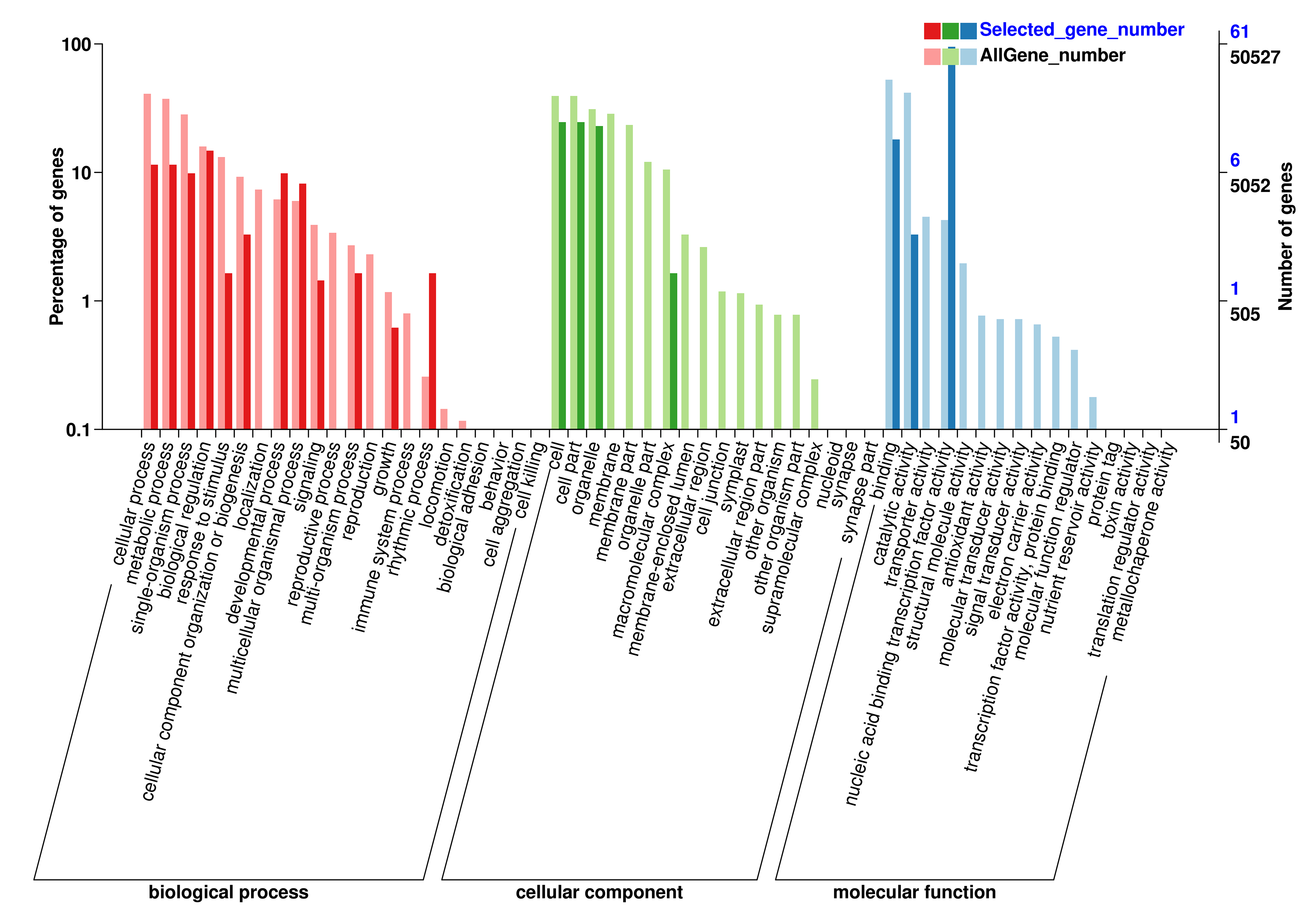

Supplement: Supplementary Figure S1 — Multiple sequence alignment of TCP proteins in Ma bamboo. [file Data_Sheet_1.ZIP › Supplementary materials/Figure S3 GO annotation results of all differential genes and TCP members in the transcriptome.jpg]

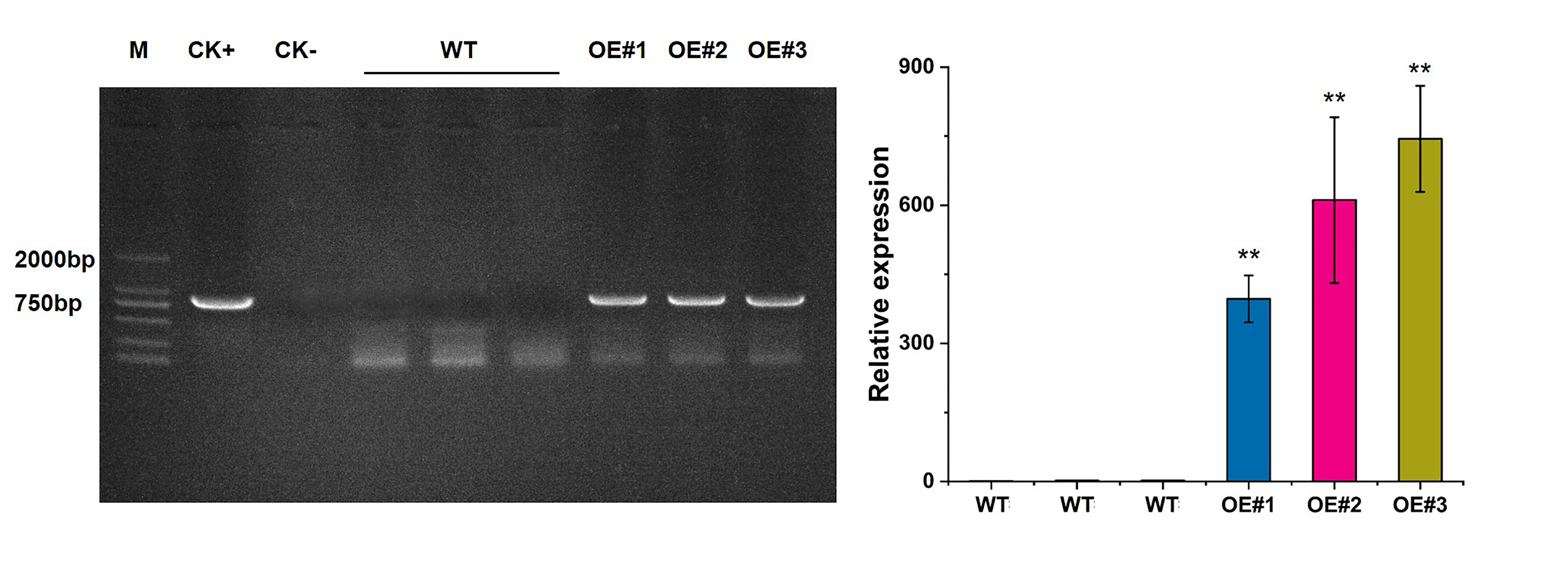

Supplement: Supplementary Figure S1 — Multiple sequence alignment of TCP proteins in Ma bamboo. [file Data_Sheet_1.ZIP › Supplementary materials/Figure S4 The results of transgene identification and expression detection.jpg]
